# Neurohypophysial Hormones Associated with Osmotic Challenges in the Brain and Pituitary of the Euryhaline Black Porgy, *Acanthopagrus schlegelii*

**DOI:** 10.3390/cells10113086

**Published:** 2021-11-09

**Authors:** Adimoolam Aruna, Chien-Ju Lin, Ganesan Nagarajan, Ching-Fong Chang

**Affiliations:** 1 Department of Aquaculture, National Taiwan Ocean University, Keelung 20224, Taiwan; n.aruna@yahoo.com; 2 Department of Aquaculture, National Pingtung University of Science and Technology, Pingtung 91230, Taiwan; X747@g4e.npust.edu.tw; 3 Department of Basic Sciences, PYD, King Faisal University, Al Hofuf 31982, Saudi Arabia; 4 Center of Excellence for the Oceans, National Taiwan Ocean University, Keelung 20224, Taiwan

**Keywords:** *arginine vasotocin*, *isotocin*, arginine vasotocin receptor, isotocin receptor, osmotic stress, homeostasis, fish

## Abstract

Our study showed differential expression of the *arginine vasotocin* (*avt*)/*isotocin* (*it*) in the brain and pituitary gland of the euryhaline black porgy (*Acanthopagrus schlegelii*) during osmotic stress. A decrease in serum osmolality and increased cortisol levels were observed after acute transfer from seawater (SW) to freshwater (FW). The increased expressions of *avt*, *avt receptor* (*avtr*: *v1a*), and *isotocin receptor* (*itr*: *itr1)* transcripts on day 1 and *it* and *itr* transcripts on days 7 and 30 were found in the brains and pituitary glands of FW fish. Increased levels of *avt* mRNA in the diencephalon and *avtr* mRNA in the pituitary together with serum cortisol on day 1 of FW exposure indicated activation of the hypothalamic–pituitary–interrenal (HPI) axis. The expression levels of *avtr* and *itr* after FW transfer were increased in the pituitary on days 7 and 30. Furthermore, *in situ* hybridization demonstrated spatially differential expression of *avt* and *itr* transcripts in nucleus preopticus parvocellularis of pars gigantocellularis (PMgc), magnocellularis (PMmc), and parvocellularis (PMpc) of the preoptic area (POA). Positive signals for *avt* and *it* were highly abundant in PMpc after FW exposure. The data suggest involvement of neurohypophysial hormones in the brain (telencephalon and diencephalon) and pituitary for osmotic stress.

## 1. Introduction

The neurohypophysial peptides (nonapeptides) *arginine vasotocin* (*avt*) and *isotocin* (*it*) are hormones that regulate homeostasis in teleosteans [1]. They belong to the arginine vasopressin-oxytocin family of peptides [2,3]. The neurohypophysial hormones are active neuromodulators in the central nervous system [4,5,6]. The *avt* and *it* hormones play versatile roles in teleosts, including the maintenance of the equilibrium of salt, fluid balance, endocrine secretion, reproduction, cardiovascular function, vocalization, and social behaviors [4,7,8,9,10]. Many of the physiological actions of *avt* and arginine vasopressin (*AVP*) are common throughout the vertebrate series [11]. The *avt* and *it* genes are produced in the magnocellular and parvocellular neurons of the preoptic nucleus [10,12,13], and their axons extend to the posterior neurohypophysis of the pituitary gland [6,14,15,16]. The functionality of these extra-hypothalamic projections was confirmed by the presence of *avt* receptors (*avtr*: *v1a*, *v1b*, *v2*) and/or *it* receptors (*itr*: *itr1*, *itr2*) in the pituitary of teleosts [6,14,15,17,18].

The synthesis and release of *avt* and *it* in responding to the stressors of the environment, osmoregulation, and biology were demonstrated in teleosts [19,20,21,22,23,24,25,26,27,28]. Changes in Avt and It concentrations in the hypothalamus, pituitary, and circulation blood have been observed in some teleosts under different stressors, such as confinement, disturbance, high density, food deprivation, or osmotic change. However, the production of Avt and It depends on the kind of stress stimulation [29].

In addition, *avt* and *it* are assumed to be important in immediate and long-term acclimation/adaptation to the changes of environmental salinity [29,30,31]. Goodson and Bass (2001) [10] found that brain *avt* acts on *avtr* to regulate behaviors associated with sociality and reproduction. These *in vitro* studies showed that the neurohypophysial hormones Avt and It exert their effects through specific receptors [32,33].

In this study, type 1 (*v1a*) *avtr* was concentrated to study expression in the brain and pituitary gland during osmotic stress. Of course, future studies should analyze *avtrv1b* and *avtrv2* as well. Interestingly, Avt could also activate *itr* [14]. Expression of *itr* in the brain, intestine, bladder, skeletal muscle, lateral line, gill, and kidney seems to imply that these receptors may act as mediators for a variety of physiological functions [14]. There are few studies related to the acclimation of freshwater (FW) and seawater (SW), and the specific roles and localization of *avt*, *avtr*, *it*, and *itr* in the brain during SW and FW acclimation in fish [15,16,34,35,36,37].

Black porgy (*Acanthopagrus schlegelii*) is a marine hermaphroditic protandrous fish with a male phase in the first two years after hatching. It then switches to a female phase after three years, or later [38,39]. Black porgy is an euryhaline marine teleost, and it can live in a wide range of water salinities. This makes black porgy an attractive aquatic animal model for physiological and endocrine studies during osmotice challenge [40,41]. In this study, we investigated the time-dependent expression levels of *avt, it*, and their receptors (*avtr*: *v1a*; *itr*: *itr1*) during transfer of fish into FW in juvenile male black porgy. We thus measured the serum osmolality and cortisol levels in black porgy on day 1, day 7, and day 30 to monitor the osmoregulatory stress. Neurohypophysial hormones and their receptors were cloned, and phylogenetic analyses of these receptors were conducted. We further used mRNA expression to localize *avt* and *it* in the brain. Quantitative real-time PCR analysis (Q-PCR) was used to assess the *avtr* (*v1a*) and *itr* (*itr1*) transcripts in the pituitary during SW and FW acclimation on day 1. *In situ* hybridization confirmed these Q-PCR day 1 results.

## 2. Materials and Methods

### 2.1. Experimental Fish

Black porgy (all male, 6 to 7 months old; *n* = 72; body mass = 17.69 ± 0.59 g, body length = 9.96 ± 0.37 cm) were kept in seawater (SW) with a natural light system in the university’s aquarium (month: October; longitude: 25.08′58.8″ N, latitude: 121.46′25.5″ E; water temperatures ranged from 23 to 27 °C). The black porgy is a marine euryhaline teleost. The fish can survive in a diluted seawater environment even in freshwater (FW). This is why we selected black porgy as an experimental model fish. The fish were given pelleted dry feed *ad libitum*. Black porgies were decapitated after being sedated with 2-phenoxyethanol for sample collection. Samples of the pituitary gland and other brain tissues (telencephalon and diencephalon) were taken and quickly frozen in liquid N_2_. The experiments were performed out according to the principles and procedures approved by the National Taiwan Ocean University’s Institutional Animal Care and Use Committee (# 99026).

### 2.2. Experimental Design

Fish (*n* = 72) were randomly divided into two groups and maintained in SW (*n* = 36) or FW (*n* = 36) to further characterize the endocrine alterations in the brain and pituitary in response to an acute osmotic stress. Fish (*n* = 12 per group) were transferred to the three distinct FW-containing tanks after an initial acclimatization period of 60 days (SW–FW transfer, FW fish). SW fish (*n* = 12 per group) were also transferred to a separate three SW tanks as a control (SW–SW transfer, SW fish).

Samples were collected on day 1, day 7, and day 30 (*n* = 8 in each group) after the transfer: These samples included blood; telencephalon (prosencephalon, including the olfactory bulb, telencephalon, and part of the preoptic area located between the anterior commissure and the optic chiasm); diencephalon (mostly the diencephalon, including the thalamus, epithalamus, subthalamus, and hypothalamus, as described previously [42]); and the pituitary gland. RNA extraction was conducted after the samples were frozen in liquid nitrogen and kept at −80 °C. For *in situ* hybridization, brain tissue with preoptic area (POA) was collected on day 1 (*n* = 4 in each group) and fixed in 4% paraformaldehyde in phosphate buffered saline. The PBS used 8 g of NaCl, 200 mg of KCl, 1.44 g of Na_2_HPO_4_, and 245 mg of KH_2_PO_4_ in 800 mL of distilled water at pH 7.4.

### 2.3. Analysis of Serum Cortisol by Enzyme Immunoassay (EIA)

A plastic syringe was used to collect blood from the caudal vasculature of the SW and FW groups (day 1, day 7 and day 30, *n* = 8 in each group) in Eppendorf tubes. Centrifugation at 8000× *g* for 5 min at 4 °C yielded serum that was then stored in a freezer until further use. The cortisol EIA kit (Cayman Chemical, Ann Arbor, MI, USA) was used to measure cortisol levels. Three milliliters of diethyl ether was used to extract the serum samples. The supernatant was maintained at 40 °C in a water bath for evaporation of ether after extraction and then stored at a freezer until further analysis. Then, cortisol extracts were re-suspended in PBS buffer. We established a parallelism between the standard curve and a serial dilution of the extracted solution. The cortisol concentrations (ng/mL) in the blood could then be calculated.

### 2.4. Analysis of Serum Osmolality with a Vapor Pressure Osmometer

We examined serum osmolality with a vapor pressure osmometer (Wescor Inc., Logan, UT, USA). The serum osmolality values of the SW and FW fish on days 1, 7, and 30 (*n* = 8 in each group) are expressed as mOsm/kg.

### 2.5. RNA Extraction, First Strand cDNA Synthesis, and Cloning

TRIzol^®^ (Gibco BRL, Grand Island, NY, USA) was used to isolate RNA from the telencephalon, diencephalon, and pituitary according to the manufacturer’s protocol. RNA pellets were dissolved in RNase-free water after total RNA was precipitated in ethanol. The RNA was examined by spectrophotometry and electrophoresis on an agarose gel to check the quantity and integrity. Single-stranded cDNA was constructed using Invitrogen reagents (Invitrogen, Carlsbad, CA, USA). In a 20 µL reaction volume, 4 µg of total RNA was reverse transcribed into first-strand cDNA using oligo (dT)_12–18_ primers and SuperScript II reverse transcriptase (Gibco BRL) under the following incubation conditions: 42 °C for 60 min, 37 °C for 15 min, and 70 °C for 15 min. The resulting cDNA was a template for PCR amplification of the gene investigation. 

The genes *avt*, *avtr*, *it*, and *itr* were cloned from the cDNA of the black porgy brain. The CLUSTAL X program (version 1.81; Conway Institute UCD, Dublin, Ireland) was used to create several alignments of the published sequences of the target genes to obtain the conserved regions: These were then used to generate the primers (Table 1). PCR reactions were performed with 10X reaction buffer (2.5 µL in 200 mM Tris-HCl, pH 8.4), 500 mM KCl, 10 mM dNTP (1 μL), 2 mM MgCl_2_ (1 µL), 0.5 μL of each 10 µM forward and reverse primer (Mission Biotech Co., Ltd., Taipei, Taiwan), cDNA (1 μL), and superscript enzyme (0.2 μL) (Invitrogen) in a 25 µL final volume. The PCR used 94 °C (5 min), 35 cycles of 94 °C (30 s), 50 °C (30 s), 72 °C (30 s), and 72 °C (10 min) (Applied Biosystems, Walthan, MA, USA). The PCR products were examined by electrophoresis on an agarose gel visualized by staining with ethidium bromide. A Gel-M^TM^ Gel Extraction System Kit (Bio 101; Viogene, La Jolla, CA, USA) cloned DNA fragments into the pGEM^®^-T Easy Vector (Promega, Madison, WI, USA) and transformed into *Escherichia coli* competent cells. The culture was grown using X-Gal/IPTG ampicillin agar plates. After 15–20 h, white colonies were chosen from the plates and cultured in LB/ampicillin liquid media. Plasmids with the inserts were sequenced by a dye-terminator cycle-sequencing kit (Perkin Elmer, Foster City, CA, USA) and applied to BLAST to compare the sequences accessible in the NCBI database.

### 2.6. Phylogenetic Analysis of itr and avtr

The encoding sequences of *itr* and *avtr* cloned from black porgy were aligned with related neurohypophysial hormone receptor amino acid sequences of other fishes which were retrieved from NCBI database. Multiple sequence alignments of nonapeptide receptors amino acid sequences were generated using MUSCLE, included in MEGA version 5.05. Phylogenetic analyses were conducted based on neighbor-joining (NJ) method with a best-fit Jones–Taylor–Thornton+Gamma (JTT+G) amino acid substitutions model in MEGA5.05 software (Pennsylvania, State University, PA, USA). Statistical support for the NJ tree was evaluated by 1000 bootstrapping replicates.

### 2.7. Quantification of avt, avtr, it, and itr by Quantitative Real-Time PCR Analysis

The mRNA levels of *avt* (GenBank accession number: MZ816922), *avtr* (GenBank accession number: MZ816921), *it* (GenBank accession number: MZ816923), and *itr* (GenBank accession number: MZ816924) were analyzed by quantitative PCR (Q-PCR) using a iQ^TM^ Multicolor Real Time-PCR Detection system (Bio-Rad Co., Hercules, CA, USA). The primers of Q-PCR for *avt*, *avtr*, *it*, and *itr* were designed according to the primer expression software (Applied Biosystems) (Table 1). Meanwhile, *beta actin* and *glyceraldehyde-3-phosphate dehydrogenase* (*gapdh*, GenBank no. DQ399798) were employed as control genes. There was a significant difference in *beta actin* between SW and FW groups. There were no significant differences in the *gapdh* transcripts in response to the salinity treatment (Supplementary Materials Figure S1). Thus, *gapdh* was used as an internal control. A serial dilution of plasmid DNA containing amplified fragments of the target genes was generated (1 µg, 10^−1^ µg, 10^−2^ µg up to 10^−10^ µg). This used a Q-PCR machine (iQ^TM^ Multicolor Real Time-PCR Detection System; Bio-Rad Co.) for gene quantification of the standards, samples, and control using iQ^TM^ SYBR green (Bio-Rad) as a dsDNA minor-groove binding dye. The primers included a forward primer and a reverse primer (3 µM of *avt* and *it*, 5 µM of *avtr* and *itr*) from Mission Biotech Co., Ltd. Each sample was run twice, and a control PCR experiment was performed for each gene. The standard curve and a log (transcript concentration) versus CT curve were generated. Here, CT is the calculated fractional cycle number at which the PCR-fluorescence product was detectable above a threshold. The standard correlations for *avt*, *avtr*, *it*, and *itr* were −0.997, −0.995, −0.999, and −0.990, respectively.

### 2.8. In Situ Hybridization

The *avt*, *avtr*, *it*, and *itr* transcripts were located in the black porgy’s brain using in situ hybridization. The neuroanatomic sections focused on the nucleus of the preoptic area: nucleus preopticus parvocellularis pars gigantocellularis, PMgc; nucleus preopticus parvocellularis pars magnocellularis, PMmc; and nucleus preopticus parvocellularis pars parvocellularis, PMpc. The tissues were embedded in paraffin after being fixed in 4% paraformaldehyde in PBS for 15–20 h at 4 °C. The paraffin sections (5 µm) of the preoptic area (POA) and sagittal sections of the pituitary were on TESPA-treated slides (3-aminopropyltriethoxysilane, Sigma, St. Louis, MO, USA).

The specific PCR product of the target gene from the plasmid DNA carrying the interest genes in the vector (pGEM-T Easy) was generated with 50 U DNA polymerase (New England Biolabs, Ipswich, MA, USA) for DNA amplification with *in situ* hybridization primers (Table 1). A kit (PCR-Advanced Clean Up Kit, Viogene) was used to purify the PCR products. This purified DNA was applied as a template for *in vitro* transcription. T7 and T3 polymerase (Promega, Madison, WI, USA) were used to prepare respective sense and anti-sense riboprobes of *avt*, *avtr*, *it*, and *itr*. DNA templates (1 µg) were incubated (37 °C for 3 h) in a thermocycler (Applied Biosystems) for *in vitro* transcription. The reaction solution contained transcription buffer (5×), 0.1 M dithiothreitol (DTT), a DIG-rNTP mix (10×) (Roche, Penzberg, Germany), RNase inhibitor (40 U/µL) (Promega), and T7 or T3 RNA polymerase (20 U/µL). This samples were then each added to sterile DEPC H_2_O to make a final volume of 20 µL. The extra template was removed by digesting with 4 µL of DNase I (10 U/µL) at 37 °C for 30 min. After incubation, the RNA probes were precipitated overnight with 2.5 µL of LiCl (4 M) and 75 µL of isopropanol at −80 °C. The pellets were collected after centrifugation (10,000× *g*, 4 °C for 30 min) and then re-suspended in RNase inhibitor (2 µL) and sterile DEPC H_2_O (98 µL). We prepared an RNA probe according to previous studies [43,44].

**Figure 1:**
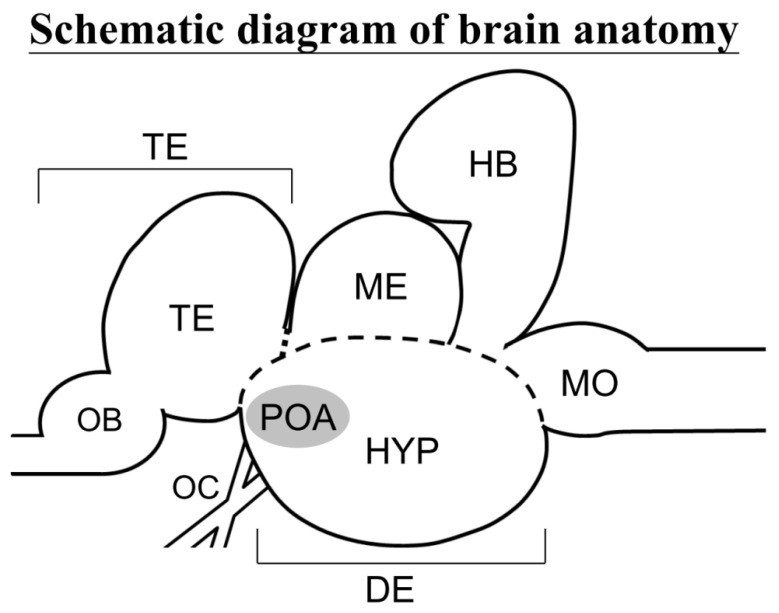
A schematic diagram showing the lateral view of a black porgy brain. DE, diencephalon; HB, hindbrain; HYP, hypothalamus; ME, mesencephalon; MO, medulla oblongata; OB, olfactory bulb; OC, optic chiasm; POA, preoptic area; and TE, telencephalon.

The sections were rehydrated (from 100% to 50% ethanol) and rinsed with PBS for multiple times. The sections were then added to proteinase K (10 µg/mL in PBS) for 8–10 min at about 25 °C before being rinsed with 0.1% Tween-20 in PBS (PBT). The sections were prehybridized (68 °C for 1 h) in prehybridization buffer and then hybridized overnight in a hybridization buffer (50% formamide, 5× SSC, 500 µg/mL tRNA, 50 µg/mL heparin and 0.1% Tween-20) with digoxigenin-labeled RNA sense or antisense probes (1 µg/mL). The sections were warmed to about 25 °C (10 min), rinsed twice with buffer containing 25% formamide, 1× SCC, and 0.1% Tween-20 (60 °C, 15 min); they were then blocked with 2% blocking reagent (Roche); 2% normal goat serum in PBT) at about 25 °C (1 h). The sections were then reacted with an alkaline phosphatase-conjugated sheep anti-digoxigenin antibody (Roche) (diluted 1:2000 in 2% blocking reagent) for 15–20 h (4 °C). The hybridization signals were visualized via NTMT (100 mM NaCl, 100 mM Tris-HCl, 50 mM Mg_2_Cl, and 0.1% Tween-20) and NBT/BCIP (nitro blue tetrazolium/5-bromo-4-chloro-indolyl-phosphate) staining. The neuroanatomical structures of the brain area in black porgy (Figure 1) were identified using the brain atlas of gilthead sea bream [45] and our previous study in black porgy [46].

### 2.9. Data Analysis

To examine the difference between the control (SW) and experimental groups (FW) on different time courses (day 1, day 7, and day 30), two-way analysis of variance (ANOVA) was conducted and followed by a Tukey’s post hoc test using a statistical tool for the social sciences (SPSS). A statistically significant value of *p* < 0.05 was used.

## 3. Results

### 3.1. Sequence Analysis of it, itr, avt, and avtr, and Phylogenetic Tree of Neurohypophysial Hormone Receptors

**Figure 2:**
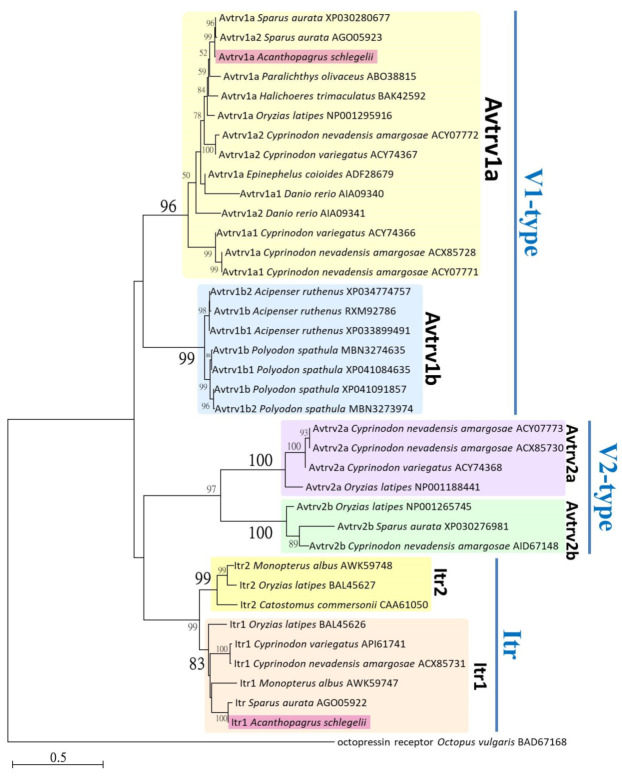
A neighbor-joining (NJ) phylogenetic tree of neurohypophysial hormone receptor amino acids. The phylogenetic tree was constructed based on the alignment of 38 partial amino acid sequences of Avtr and Itr from selected fishes using the neighbor-joining method with pairwise deletion and 1000 bootstrap replicates. The number shown at each branch node indicates the bootstrap value (%); only values and branching above 50% are shown. This tree was rooted using the sequence of octopressin receptor from an octopus and shows major branches of two V1-type receptors (Avtrv1a and Avtrv1b), two V2-type receptors (Avtrv2a and Avtrv2b), and two It receptors (Itr1 and Itr2). The cloned sequences from black porgy were grouped into Avtrv1a and Itr1, and are illustrated. Scientific names of fishes and GenBank accession numbers of sequences are shown in figure.

Multiple sequence alignments of *it*, *itr*, *avt*, and *avtr* of black porgy and other fish species are shown in the Supplementary Materials figures (Figures S2–S5). The percentages of identity for black porgy genes compared to those of other fish species are 88.58–96.33% for *it*, 95.36–96.50% for *itr*, 87.23–96.54% for *avt*, and 89.05–92.51% for *avtr* (Figures S2–S5). A total of 38 amino acid sequences of representative species were used for phylogenetic analysis. The bootstrapping values below 50% are not shown in the Figure 2. Phylogenetic analyses were used to estimate the evolutionary relationship between the Avtr and Itr sequences of black porgy from other noanpeptide receptor sequences in fishes. Based on alignment of partial amino acid sequences, and by taking the sequence of octopressin receptor from octopus (*Octopus vulgaris*) as an outgroup, an NJ phylogenetic tree was constructed. The noanpeptide receptor sequences clustered into major branches of two V1-type receptors (Avtrv1a and Avtrv1b), two V2-type receptors (Avtrv2a and Avtrv2b), and two It receptors (Itr1 and Itr2). The encoding Avtr and Itr sequences of black porgy cloned and used in this study were clustered with the clad of Avtrv1a and Itr1, respectively. Tree topology and sequences of black porgy are illustrated in Figure 2.

### 3.2. Serum Osmolality and Cortisol

**Figure 3:**
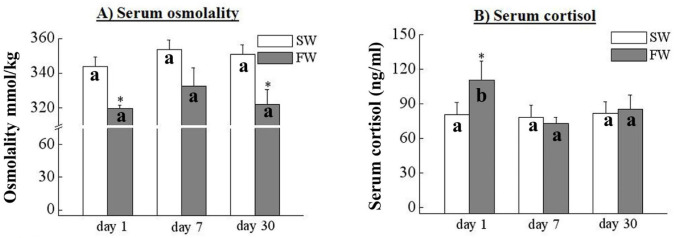
(**A**) Analysis of the serum osmolality and (**B**) serum cortisol in FW and SW black porgy on days 1, 7, and 30 (*n* = 8, in each group). The results are expressed as mean ± SEM (standard error of mean). Different letters indicate significant a difference between sampling points with the same treatment; asterisks (*) show differences between groups at the same time points (two-way ANOVA followed by Tukey’s test, *p* < 0.05).

The serum osmolality levels were significantly decreased on day 1 and 30 in the FW fish compared to the SW control (Figure 3A). The osmolality was not statistically different between SW and FW (Figure 3A). The serum cortisol levels were significantly increased in the FW fish on day 1 compared to the SW control (Figure 3B); there were no differences in serum cortisol levels on day 7 and day 30 in the FW fish compared to the SW control (Figure 3B).

### 3.3. The Expression of avt Transcripts in the Brain

**Figure 4:**
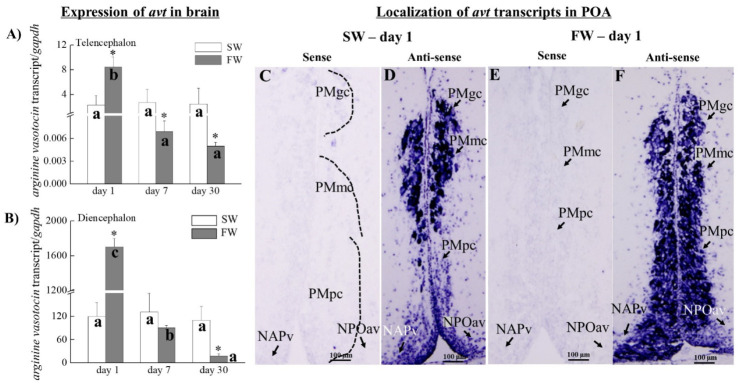
Q-PCR was performed to analyze the transcripts of *avt* in the telencephalon (**A**) and diencephalon (**B**) of SW and FW black porgies on day 1, day 7, and day 30 (*n* = 8 in each group). Gene expression was normalized to the control (*gapdh*) gene. The results are expressed as mean ± SEM. Different letters indicate a significant difference between sampling points with the same treatment; asterisks (*) show differences between groups at the same time point (two-way ANOVA followed by Tukey’s test, *p* < 0.05). (**C**–**F**) Localization of *avt* transcripts in SW and FW black porgies’ preoptic areas (POA) on day 1 (*n* = 4 in each group). The transcripts of *avt* (**D**,**F**) were detected in both SW and FW fish. Furthermore, the *avt* transcripts were increased in PMmc and PMpc of the FW fish (**F**) compared to the SW fish (**D**). No difference was found between SW (**D**) and FW (**F**) fish in PMgc on day 1. One of the representative fish from each group was used for the histological data. There were no signals detected in the SW (**C**) and FW (**E**) fish when using the respective control sense probes of *avt* on day 1. PMgc: preopticus parvocellularis of pars gigantocellularis; PMmc: preopticus parvocellularis of pars magnocellularis; PMpc: preopticus parvocellularis of pars parvocellularis; NAPv: nucleus anterioris periventricularis; and NPOav: anteroventralis nucleus preopticus.

The *avt* transcripts in the telencephalon (increased by 3.6-fold; Figure 4A) and diencephalon (increased by 15-fold; Figure 4B) were significantly increased in the FW fish on day 1 compared to SW fish. The *avt* transcripts in the telencephalon (Figure 4A) were significantly decreased on day 7 (15-fold) and 30 (10-fold) in the FW compared to the SW fish. The diencephalon did not have a difference between the SW and FW fish on day 7 (Figure 4B). Among the FW fish groups, the *avt* mRNA was significantly decreased on day 7 and day 30 compared to day 1 in the telencephalon and diencephalon (Figure 4A,B).

### 3.4. Localization of avt Transcripts in the SW and FW Black Porgies’ Preoptic Areas

*In situ* hybridization was performed to localize the *avt* transcripts (Figure 4C–F) in the SW and FW black porgies’ POAs. Transcripts from *avt* were detected in the POAs of both SW and FW black porgies. Furthermore, the levels of the *avt* transcripts were increased in the preopticus parvocellularis of pars magnocellularis (PMmc) (Figure 4F) and the preopticus parvocellularis of pars parvocellularis (PMpc) (Figure 4F) of the FW fish on day 1 compared to the SW fish (Figure 4D). The preopticus parvocellularis of pars gigantocellularis (PMgc) revealed no differences between the SW (Figure 4D) and FW fish (Figure 4F) on day 1. There were no signals in the SW and FW fish when using the respective control sense probes of *avt* on day 1 (Figure 4C,E).

### 3.5. The Expression of avtr Transcripts in the Pituitary

**Figure 5:**
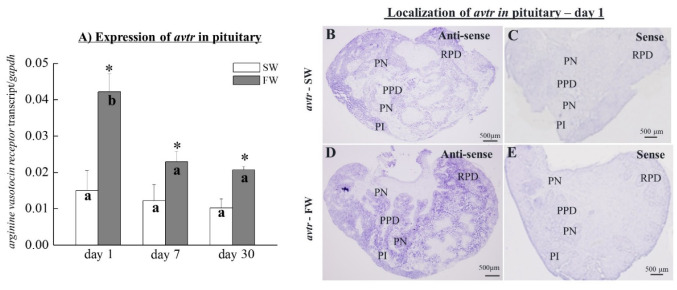
(**A**) Q-PCR analyzed the transcripts of *avtr* in the pituitaries of SW and FW black porgies on day 1, day 7, and day 30 (*n* = 8 in each group). Gene expression was normalized to the control (*gapdh*) gene. The results are expressed as mean ± SEM. Different letters indicate a significant difference between sampling points with the same treatment; asterisks (*) show differences between groups at the same time point; (two-way ANOVA followed by Tukey’s test, *p* < 0.05). (**B**–**E**) *In situ* hybridization of *avtr* in black porgy pituitary (*n* = 4). The *avtr* transcripts were detected in the RPD of the FW (**D**) pituitary compared to the SW fish (**B**). There were no signals detected in the SW and FW fish when using the respective control sense probes of *avtr* on day 1 (**C**,**E**). RPD: rostral pars distalis; PPD: proximal pars distalis; PI: pars intermedia; and PN: pars nervosa.

The transcripts of *avtr* were detected in the pituitary (Figure 5A). The *avtr* transcripts were significantly increased on days 1, 7, and 30 in the pituitary of the FW fish when compared to the SW controls (increased by 4.2-fold, 2.2-fold, and 1.8-fold on days 1, 7, and 30, respectively; Figure 5A). Among the FW fish groups, the *avtr* mRNA was significantly decreased on day 7 and day 30 compared to the FW fish on day 1 (Figure 5A).

### 3.6. Localization of avtr Transcripts in Black Porgy Pituitary Glands

The *avtr* transcripts were detected via *in situ* hybridization in the RPD at both SW (Figure 5B) and FW (Figure 5D) fish. The *avtr* transcripts were notably increased in RPD in FW fish (Figure 5D) compared to the SW fish (Figure 5B). There were no signals detected for the control sense probes of *avtr* (Figure 5C,E).

### 3.7. The Expression of it Transcripts in the Brain

**Figure 6:**
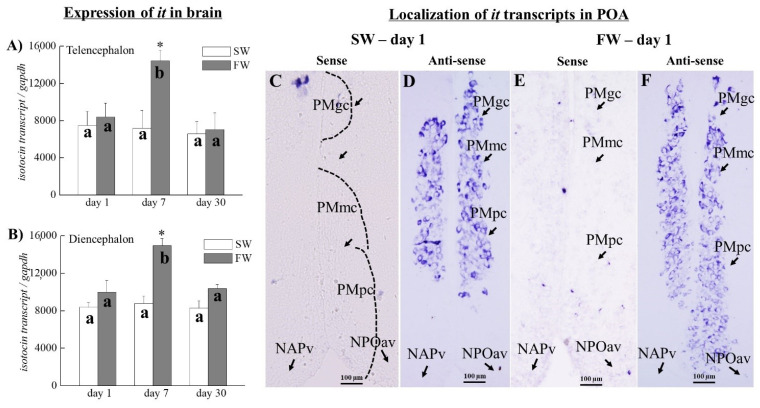
(**A**,**B**) Q-PCR was performed to analyze the transcripts of *it* in telencephalon (**A**) and diencephalon (**B**) of SW and FW black porgies on day 1, day 7, and day 30 (*n* = 8 in each group). Gene expression was normalized to the control (*gapdh*) gene. The results are expressed as mean ± SEM. Different letters indicate a significant difference between sampling points with the same treatment; asterisks (*) show differences between groups at the same time point (two-way ANOVA followed by Tukey’s test, *p* < 0.05). (**C**–**F**) The localization study (*n* = 4 in each group) of *it* transcripts revealed them in the preoptic areas (POAs) of the SW (**D**) and FW (**F**) black porgies on day 1. There were no differences in the *it* transcripts in PMgc and PMmc, but these increased in PMpc on day 1 of FW (**F**) compared to the SW control (**D**). One of the representative fish from each group is presented for histological data. There were no signals detected in the SW and FW fish when using the *it* respective control sense probes on day 1 (**C**,**E**). PMgc: preopticus parvocellularis of pars gigantocellularis; PMmc: preopticus parvocellularis of pars magnocellularis; and PMpc: preopticus parvocellularis of pars parvocellularis. NAPv: nucleus anterioris periventrcularis; and NPOav: anteroventralis nucleus preopticus.

There were no significant differences in the transcripts of *it* in the telencephalon and diencephalon between the SW and FW fish on days 1 and 30 (Figure 6A,B). However, the *it* transcripts in the telencephalon (increased by 2-fold) and diencephalon (increased by 1.71-fold) were significantly increased in the FW fish on day 7 compared to the SW fish (Figure 6A,B). Among the FW groups, *it* mRNA expression was significantly increased on day 7 compared to the FW fish on day 1 and day 30 in the telencephalon and diencephalon; there were no differences in the *it* transcripts among the SW fish on day 1, day 7, and day 30 (Figure 6A,B).

### 3.8. Localization of it Transcripts in the SW and FW Black Porgies’ Preoptic Areas

Transcripts of *it* (Figure 6D,F) were detected in the preoptic areas of the SW and FW black porgies on day 1 (Figure 6D,F). There were no differences in the intensity of *it* transcripts in the PMgc and PMmc, but they slightly increased in the PMpc of the FW fish on day 1 (Figure 6F) compared to the PMpc of the SW fish (Figure 6D). There were no signals in the SW and FW fish when using the *it* control sense probes on day 1 (Figure 6C,E).

### 3.9. The Expression of itr Transcripts in the Pituitary

**Figure 7:**
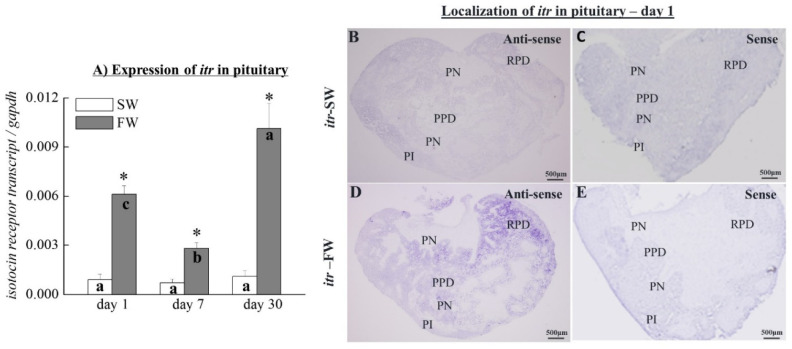
(**A**) Q-PCR analyzed the transcripts of *itr* (**A**) in the pituitary glands of SW and FW black porgies on day 1, day 7, and day 30 (*n* = 8, in each group). Gene expression was normalized to the control (*gapdh*) gene. The results are expressed as mean ± SEM. Different letters indicate a significant difference between sampling points with the same treatment; asterisks (*) show differences between groups at the same time point (two-way ANOVA followed by Tukey’s test, *p* < 0.05). (**B**–**E**) The localization study of *itr* transcripts (*n* = 4 in each group; revealed them in the pituitary glands of the SW (**B**) and FW (**D**) black porgies on day 1. There were no signals detected in the SW and FW fish when using the respective control sense probes of *itr* on day 1 (**C**,**E**). RPD: rostral pars distalis; PPD: proximal pars distalis; aNH: anterior neurohypophysis; pNH: posterior neurohypophysis.

The transcripts of *itr* in the pituitary were increased by the osmotic stress on day 1, day 7, and day 30 (by 6.6-fold, 4-fold, and 9-fold, respectively; Figure 7A) compared to control SW fish. The *itr* mRNA increased significantly on day 1 and day 30 in FW fish compared to FW fish on day 7 in the pituitary (Figure 7A).

### 3.10. Localization of itr Transcripts in the SW and FW Black Porgies’ Pituitary Glands

The *in situ* hybridization showed that transcripts of *itr* were detected in the pituitary of the SW (Figure 7B) and FW (Figure 7D) black porgy on day 1. The *itr* transcripts were increased in the RPD on day 1 of the FW fish (Figure 7D) compared to SW fish (Figure 7B). There were no signals detected in the SW and FW fish when using *itr* control sense probes on day 1 (Figure 7C,E).

## 4. Discussion

We developed an experimental system to understand the molecular and cell level associations of neurohypophysial peptides (*avt*, *it*) and their receptors (*avtr*: *v1a*, *itr*: *itr1*) in the brains and pituitaries of black porgies during SW and FW acclimation by Q-PCR and *in situ* hybridization analysis. There is limited information on the localization of *avt*, *it*, and their receptors in the brain (telencephalon and diencephalon) and pituitary over a serial time course exposure to osmotic stress (e.g., day 1 [acute], day 7 [acclimation], and day 30 [chronic]).

A single cDNA encoding *avt*, *avtr*, *it*, and *itr* was cloned and sequenced in the brain of a black porgy in this study. The nucleotide sequences of *avt*, *avtr*, *it*, and *itr* showed homology at multiple nucleotide locations that are linked to the *Sparus aurata* sequence [47]. According to the comparatively phylogenetic analysis, Avtr and Itr sequences clustered into two V1-type receptors (Avtrv1a and Avtrv1b) and two V2-type receptors (Avtrv2a and Avtrv2b), and two Itr (Itr1 and Itr 2) (Figure 2). Our black porgy Avtr and Itr were clustered with the clad of Avtrv1a and Itr1 (Figure 2). Our data clearly provided a more specific phylogenetic tree among various fish species as compared to previous studies [15,48,49].

The serum osmolality levels were significantly decreased within the FW black porgy on day 1 and 30. In the SW fish, serum osmolality was significantly higher than in the FW fish, as reported in previous studies of rabbitfish (*Siganus rivulants*) [50], black porgy [40], and spotted tail goby (*Synechogobius ommaturus*) [51]. The study by Kammerer et al. (2010) [52] showed that plasma cortisol increased rapidly within 3 h of SW transfer and remained elevated for three days. They then returned to basal levels similar to gilthead sea bream (*Sparus aurata*) in FW [37]. Studies of *Oreochromis mossambicus* suggested that serum cortisol levels increased when the tilapia were transferred from SW to FW [52,53]. The level of serum cortisol significantly increased on day 1 and returned to normal on days 7 and 30, suggesting that the black porgy only needs a brief time interval (less than 7 days) to acclimate osmotic stress.

At various time points following FW transfer, we characterized the *avt* and *it* mRNA in the brain and the *avtr* (*v1a*) and *itr* mRNA (*itr1*) in the pituitary. We found increased levels of *avt* mRNA in the diencephalon and telencephalon and *avtr* and *itr* mRNA in the pituitary, together with an increased serum cortisol level on day 1 in the FW fish. This implies that brain *avt* undergoes action through the pituitary *avtr* or *itr* and releases the final product of the HPI axis, i.e., cortisol. This suggests that *avt* could trigger the HPI axis during osmoregulatory stress in black porgy. Avt triggers the stress response in gilthead seabream after a low dose of cortisol [35,36]. Few studies have looked at *avt* and *it* in the teleost diencephalon, telencephalon, preoptic area, and other regions outside of the preoptic nuclei and hypothalamus by Q-PCR and *in situ* hybridization in association with the social behavior or diurnal profiles [54,55,56]. Importantly, the *avt* preprohormone mRNA was found in multiple regions of the brain, such as the olfactory bulb, preoptic area, hypothalamus, and also dorsomedial, ventral, and central regions of the telencephalon, suggesting the possible involvement in the behavioral regulation in African cichlid fish (*Astatotilapia burtoni*) [56].

Endogenous cortisol influences Avt and It secretion from pituitary cells in gilthead sea bream [57]. Cadiz et al. (2015) [37] reported that the *avt* and *it* levels were progressively elevated in the pituitary collaterally with high hypothalamic *avt* and *it* expression at 24 h post-injection in a cortisol-treated group of gilthead sea bream. Elevated plasma Avt levels were found in gilthead sea bream [29] and flounder [58] exposed to a rapid osmotic challenge. Hypothalamic *avt* transcripts increased two days after transferring dogfish (*Triakis scyllium*) to high salinity water (45.5‰), [59]. The decreased *avt* mRNA expression in FW fish at days 7 and 30 may result from negative feedback of the HPI axis or an increase in cortisol content that de-sensitizes the telencephalon *avt* mRNAs in black porgy. In teleosts, corticotropin-releasing hormone (*crh*) and *avt* are synergized to stimulate *acth* release from the anterior pituitary [60]. The final product of the stress axis, cortisol, is suggested to stimulate both glucocorticoid and mineralocorticoid receptors in fish [43,44,53].

The *it* mRNA from the telencephalon and diencephalon increased on day 7 in the FW group compared to their respective baseline groups. These results revealed that the *it* mRNA may also be involved in triggering the HPI axis in black porgies after initial acclimation. This compensation may allow fish to stay in homeostasis during prolonged osmotic stress. The HPI axis’s negative feedback may have contributed to the lower serum cortisol levels. Exogenous Avt decreased the storage of pituitary It in gilthead sea bream, and *itr* expression was enhanced [48]. Compared to the corresponding controls, *avtr* and *itr* transcripts were consistently increased in the pituitary on day 1, day 7, and day 30 upon FW transfer in the current investigation. The *itr* in the pituitary could interact with glucocorticoid receptors for maintenance of homeostasis in black porgies under various osmotic stress conditions.

We found that the hybridization signals for *avt* and *it* transcripts were in the PMgc, PMmc, and PMpc of the POA in the SW and FW groups. Interestingly, the intensities of the *avt* and *it* mRNA hybridization signals were strong in the PMmc and PMpc of the black porgy POAs during FW acclimation compared to the respective SW fish on day 1. Our data suggest that the PMmc and PMpc neurons (especially the PMpc) are involved in coping with the external osmotic stress in the FW-acclimated fish. In contrast, *avt* and *it* mRNAs were expressed similarly in the PMgc during SW and FW acclimation.

A number of previous studies have reported that these nuclei are associated with distinct physiological functions. The PMgc responds to acute stress [61]; the PMmc is involved in blood pressure, osmoregulation, and also the response to acute stress [22,61]; and the PMpc nuclei are involved in stress and the release of the stress hormones [19,62]. In rainbow trout, acute confinement stress was associated with enhanced Avt production in the parvocellular neurons of the preoptic nucleus [21]. Following water deprivation, increased expression of *avt* transcripts and Avt in the paraventricular nucleus suggests that osmotic stress induces transcription of the *avt* gene in hypothalamic neurons in the quail brain [63]. A recent study found that acute air exposure stress changes *avt* and *it* expression in the hypothalamus and their receptors in the pituitary gland in gilthead sea bream [36]. Overall, our data confirm that *avt* and *it* expressed in the teleost brain indicate that these neuropeptides are modulated under multiple physiological processes [13,64].

The *avtr* and *itr* mRNA hybridization signals were detected at rostral pars distalis of the black porgy pituitary. The Avt binding sites in white suckers (*Catostomus commersoni*) have been discovered in the area occupied by corticotrope cells [65]. The V1a-type receptors were highly expressed in the RPD of the pituitary [66]. The V1b-type receptor is also found in the anterior pituitary glands of mammals [67] and avian species [68], which is where ACTH cells are found. The V1a-type receptor mRNA has been found in the anterior pituitary glands of bullfrogs [69] similar to our findings. The expression of V1 and V2-type receptors is species-specific, time-dependent, and changes based on the type of stress. There are only limited studies about the expression and localization of *itr* in the pituitary. The *itr1* and *itr2* mRNA and proteins are also expressed in rice field eels [8]. The *it* may regulate osmoregulation via *itr1*, but not *itr2* in eels [70]. The *itr1* and *itr2* immunoreactive cells are localized in different areas of the eel’s pituitary [70]. Based on the current interesting data, in the future, we should further extend the studies of osmotic stress to other types of *itr* and *avtr* in black porgy.

## 5. Conclusions

In summary, we report here the differential expression and localization of *avt*, *avtr*, *it*, and *itr* after FW transfer at different time points. We showed changes in the expression of *avt* and *it* and their receptors (*avtr*: *v1a*, *itr*: *itr1*). We also evaluated mRNA expression and localization in the brain together with serum cortisol levels during various times after exposure to osmotic stress. Expression levels of the *avt* and *it* transcripts were differentially sensitive to the external salinity and different exposure times. Furthermore, the *avt* and *it* positive neurons and the intensity of the hybridization signals increased in the PMpc of the preoptic area after FW transfer. The *avt* and *avtr*/*itr* (day 1) responded at the time of initial exposure, but only after *it* (day 7) and *avtr/itr* (day 7 and day 30) took over to respond to the osmotic stress. Our data confirm that these neurohypophysial peptides are necessary during osmotic stress and are important for homeostasis in black porgy.

## Figures and Tables

**Table 1:** Oligonucleotide primers used for specific primers for reverse transcription PCR (RT-PCR), quantitative real-time PCR (Q-PCR) analysis, and in situ hybridization. S: sense primer, AS: anti-sense primer, F: forward primer, and R: reverse primer.

Gene	Orientation Usage	Nucleotide Sequence (5′–3′)	Amplicon Size
*avt*	RT-PCR-F	ATGCCTCACTCCATGTTC	450 bp
	RT-PCR-R	CTGTCCTCTGGTGGCCACATGTAG	
	Q-PCR-F	CCCCCTGTGCGTCCTGGGACTCATC	378 bp
	Q-PCR-R	CCTCCCCGAGACAGTCAGAGTC	
	in situ-S	CCTGTGCGTCCTGGGACTCATC	344 bp
	in situ-AS	CACCCCACAGAGCTGT	
*avtr*	RT-PCR-F	GAGGTGGCCCAAATCGAGATC	896 bp
	RT-PCR-R	GGCGCCCAGCAGATAATGTAC	
	Q-PCR-F	CCTGCTGGCAATGTACAACAC	561 bp
	Q-PCR-R	GCAGCGCCGGCCATCGTTTTC	
	in situ-S	GTCGCGGATGCACCTCTTCATC	463 bp
	in situ-AS	CCCGTAGCACATCATGAGAATG	
*it*	RT-PCR-F	GCTGGGGGGACTTTGTGC	342 bp
	RT-PCR-R	CTGCAGGGTCCCATA	
	Q-PCR-F	CCGTGGGGAGCCAAAGCTTAC	307 bp
	Q-PCR-R	CAGACCACATCTGGACAAAGAA	
	in situ-S	CACATGGATGAGCCTCAGC	262 bp
	in situ-AS	GATGGGAGTCCAGCACATTATG	
*itr*	RT-PCR-F	GCGTGTTACATCTCCAACTGT	393 bp
	RT-PCR-R	GGTGTGG CCGGCCAGAT	
	Q-PCR-F	CGGAGGGAAGAGATCCATCATG	316 bp
	Q-PCR-R	GCCTTCGGATTGGCTGCTTTG	
	in situ-S	GCACCGCTGCGCAAGTGCATGTG	325
	in situ-AS	GCAGTAGCCTGAGGATGATGTC	
*gapdh*	RT-PCR-F	GGCCCCCCTGGCCAAAGT	523 bp
	RT-PCR-R	TGGGTGTCACCGATGAAG	
	Q-PCR-F	AGGCTTCCTTAATCTCAGCATAAGAT	490 bp
	Q-PCR-R	GGTGCCTGTGGCTGATGTG	

## Data Availability

Data are contained within this article. Raw data are available on request from the corresponding authors.

## Supplementary Materials

The following are available online at https://www.mdpi.com/article/10.3390/cells10113086/s1, Figure S1: The expression (*n* = 8 in each data) of internal control gene *gapdh* (*glyceraldehyde 3-phosphate dehydrogenase*, GenBank accession no: DQ399798) in black porgy acclimated to seawater (SW) and freshwater (FW). No significant difference (denoted with a) of *gapdh* transcripts was found between SW and FW fish. No significant changes (denoted with a) of *gapdh* transcripts were observed on day 1, 7, and 30. Figure S2: Multiple alignments of the nucleotide sequences of *it* (black porgy, *Acanthopagrus schlegelii*) with other teleosts. GenBank accession numbers for the nucleotide sequences are follows: gilthead sea bream (*Sparus aurata*, FR851925 *it*), European flounder (*Platichthys flesus*, AB036518 *it*), sebase clownfish (*Amphipirion sebar*, KP260906 *it*), cichlid fish (*Haplochromis burtoni*, GQ288466 *it*), multicolour rainbowfish (*Parajulis poecilepterus*, DQ073095 *it*), spotty warsse (*Notolabrus celidotus*, MK252281 *it*). The boxes represent the ATG start codon and underlines represent the stop codon. The symbols “*”, “.”, and “:” denote the identity, single nucleotide variation, and more than one nucleotide variation among fish species, respectively. Figure S3: Multiple alignments of the nucleotide sequences of *itr* (black porgy, *Acanthopagrus schlegelii*) with other teleosts. GenBank accession numbers for the nucleotide sequences are follows: gilthead sea bream (*Sparus aurata*, KC195973 *itr*), Asian swamp eel (*Monoppterus albus*, MF996357 *itr*), sheepshead minnow (*Cyprinodon variegatus*, KX061748 *itr*), amargosa pupfish (*Cyprinodon nevadensis*, GQ981415 *itr*), medaka (*Oryzias latipes*, AB646240 *itr*). The boxes represent the ATG start codon and underlines represent the stop codon. The symbols “*”, “:”, and “:” denote the identity, single nucleotide variation, and more than one nucleotide variation among fish species, respectively. Figure S4: Multiple alignments of the nucleotide sequences of *avt* (black porgy, *Acanthopagrus schlegelii*) with other teleosts. GenBank accession numbers for the nucleotide sequences are follows: gilthead sea bream (*Sparus aurata*, FR851924), orange spotted grouper (*Epinephelus coioides*, GU831571), European flounder (*Platichthys fleus*, AB036517), Chinese warasse (*Halichoeres tenuispinis*, GU212654), olive flunder (*Paralicthys olivaceus*, AB856411). The boxes represent the ATG start codon and underlines represent the stop codon. The symbols “*”, “.”, and “:” denote the identity, single nucleotide variation, and more than one nucleotide variation among fish species, respectively. Figure S5: Multiple alignments of the nucleotide sequences of *avtr* (black porgy, *Acanthopagrus schlegelii*) with other teleosts. GenBank accession numbers for the nucleotide sequences are follows: orange spotted grouper (*Epinephelus coioides*, GU929704), clown anemonefish (*Amphiprion ocellaris*, AB597955), olive flounder (*Paralicthys olivaeus*, EF451960), threespot wrasse (*Halichores trimaculatus*, AB642257), medaka (*Oryzias latipes*, NM001308987). The boxes represent the ATG start codon and underlines represent the stop codon. The symbols “*”, “.”, and “:” denote the identity, single nucleotide variation, and more than one nucleotide variation among fish species, respectively.
