# Supplementary material for: Neurohypophysial Hormones Associated with Osmotic Challenges in the Brain and Pituitary of the Euryhaline Black Porgy, Acanthopagrus schlegelii
Source: Cells. 2021 Nov 9;10(11):3086. doi: 10.3390/cells10113086 (PMC8624723; doi:10.3390/cells10113086)
Supplement: Supplementary file 1 [file cells-10-03086-s001.zip › cells-1366984 -Fig S2-2021-10-31.pdf]

```
Black porgy      ATGACTGGAGCTGCTGTGTCCGTGTGCCTTGTTTTTCTGCTGTCTGTATGTTCAGCGTGT
Haplochromis     ------------------------------------------------------GCCTGC
Sparus           ATGACTGGAGCTGCTGTGTCCGTGTGCCTTGTTTTTCTCCTGTCTGTATGTTCAGCGTGT
Parajulis        ATGACTGGAGCCTCTGTGTCCGTGTGCCTTCTTTTCCTCCTGTCCGTCTGTTCAGCATGT
Notolabrus       -------------------------GCCTTCTTTTCCTCCTATCCGTCTGTTCAGCATGT
Amphipirion      ATGACCGGAGCTGCTGTGCCCGTCTGCTTTCTTTTCCTTCTGTCTGTATGCTCAGCCTGT
Platichthys      ATGACTGGAGCCGCTGTTTCCGTGTGCCTACTTTTTCTTGTGTTTCTATGTTCAGCATGT
                                                                       ** **

Black porgy      TACATCTCTAGCTGTCCCATCGGAGGGAAGAGATCCATCATGGATGCACCGCTGCGCAAG
Haplochromis     TACATCTCCAACTGTCCAATCGGAGGAAAAAGGTCCATGATGGACGCACCCCTGCGCAAG
Sparus           TACATCTCTAACTGTCCCATCGGAGGGAAGAGATCCATCATGGATGCACCGCTACGCAAG
Parajulis        TACATCTCTAACTGTCCTATCGGGGGGAAGAGGTCAATTATGGACGCACCACAGCGCAAG
Notolabrus       TACATCTCTAACTGTCCTATCGGGGGGAAGAGGTCCATCATGGACGCACCACAGCGCAAG
Amphipirion      TACATCTCCAACTGTCCTATTGGTGGGAAGAGGTCCATCATAGATGCACCACTGCGGAAG
Platichthys      TACATCTCAAACTGTCCCATCGGCGGGAAGCGGTCCATCATGGATGCACCACTACGCAAG
                 ******** *.****** ** ** **.**..*.**.** **.** ***** *:.** ***

Black porgy      TGCATGTCGTGTGGCCCCGGAGACAGGGGCCGCTGCTTCGGCCCCAACATCTGCTGTGGG
Haplochromis     TGCATGTCGTGTGGCCCCGGAGACAGAGGCCGCTGCTTCGGGCCAAGTATCTGCTGCGGG
Sparus           TGCATGTCGTGTGGCCCCGGAGACAGGGGCCGCTGCTTCGGCCCCAGTATCTGCTGCGGG
Parajulis        TGCATGCCCTGTGGCCCTGGAGACCGGGGCCGCTGCTTTGGCCCCAATATCTGCTGCGGC
Notolabrus       TGCATGTCGTGTGGCCCTGGAGACAGGGGCCGTTGTTTCGGCCCCAGTATCTGCTGTGGA
Amphipirion      TGCATGCCCTGTGGCCCCGGAGACAAGGGCCGCTGCTTCGGCCCAGGTATCTGCTGTGGG
Platichthys      TGCATGTCCTGTGGCCCCGGAGACAGGGGCCGCTGCTTCGGCCCCGGTATCTGCTGCGGG
                 ****** * ******** ******...***** ** ** ** **... ******** **

Black porgy      GAGGGCCTCGGCTGTCTGCTGGGCTCCCCGGAAGCAGCTCACTGCGTGGAGGAGAACTAT
Haplochromis     GAAGGTCTTGGTTGCCTGCTCGGCTCCCCGGAAACAGCTCACTGTGTGGAGGAGAACTAC
Sparus           GAGGGCCTCGGTTGTCTGCTTGGCTCCCCGGAAACAGCTCACTGTGTGGAGGAGAACTAC
Parajulis        GAGGGCCTGGGCTGTTTGCTGGGCTCCCCAGAAACAGCTCACTGTTTGGAGGAGAACTAC
Notolabrus       GAGGGCCTTGGTTGCTTGCTGGGCTCCCCAGAAACAGCTCACTGCGTCGAGGAGAACTAC
Amphipirion      GAGGGTCTCGGCTGCCTGCTGGGCTCCCCAGAAACAGCTCACTGTGTGGAGGAGAACTAC
Platichthys      GAGGGTCTGGGCTGCCTGCTGGGCTCCCCAGAGACAGCTCACTGTGTGGAGGAGAACTAC
                 **.** ** ** **  **** ********.**..**********   * ***********

Black porgy      CTGCTCACCCCCTGCCAGGCGGGAGGGAGACCCTGTGGCTCTGAAGGAGGACGCTGCGCT
Haplochromis     CTGCTCACCCCCTGCCAGGCAGGAGGACGACCCTGTGGTTCTGAAGGAGGACGCTGTGCT
Sparus           CTGCTCACCCCCTGCCAGGCGGGAGGGAGACCCTGTGGATCTGAAGGAGGACGCTGCGCT
Parajulis        CTGCTCACCCCCTGCCAGGCGGGAGGGAGACCCTGTGGATCTGAGGGAGGACGCTGTGCT
Notolabrus       CTGCTCACCCCCTGCCAGGCAGGAGGGAGACCCTGCGGATCTGAGGGAGGACGCTGCGCT
Amphipirion      CTGCTGACTCCCTGCCAGGCAGGAGGACGACCCTGCGGATCTGAGGGAGGACGCTGCGCT
Platichthys      CTGCTCACCCCCTGCCACGCAGGAGGGAGACCCTGTGGATCTGAGGGAGGACGCTGTGCG
                 ***** ** ******** **.*****..******* ** *****.*********** **

Black porgy      GCTTCAGGACTCTGCTGCGATGCAGAGAGCTGCACCACAGACCAATCCTGCCTCATCGAG
Haplochromis     GCTTCAGGACTCTGCTGTGATGCAGAGAGCTGCACCACAGATCAATCCTGCCTTATCGAG
Sparus           GCTTCAGGACTCTGCTGCGATGCAGAGAGCTGCACCACAGACCAATCCTGCCTCATCGAG
Parajulis        GCCTCAGGAGTCTGCTGCGATGCAGAGAGCTGCACTACAGACCAATCCTGCCTCATGGAC
Notolabrus       GCCTCAGGAGTCTGCTGTGATGCAGAAAGCTGCACTACAGACCAATCCTGCCTCGTGGAC
Amphipirion      GCTTCAGGACTCTGCTGTGATGCAGAGAGCTGCACCACAGACCAGTCCTGCCTCATGGAG
Platichthys      GCATCAGGACTCTGCTGTGATGCAGAGAGTTGCACCACAGACCAATCCTGCCTCATCGAG
                 ** ****** ******* ********.** ***** ***** **.******** .* **

Black porgy      GAGGAGGGAGACGACCAAAGCAGCCAATCCGAAGGCAGCGACACCGGTGACATCATCCTC
Haplochromis     GAGGAAGGAGATGAACAAACCAGCCAGTTCGATGGCAGCGACCCCGGTGACATCATCCTC
Sparus           GAGGAGGGAGATGACCAAAGCAGCCAATTCGAAGGCGGTGACACCGGTGACATCATCCTC
Parajulis        GAGGAGGGCGACGACCCAACCAGCCAATTCGAAGGTGGTGACCCTGGTGACATCATCCTC
Notolabrus       GAGGAGGGCGACGACCAAACCAGCCAATTCGAAGGCAGCGACCCTGGTGACATCATCCTC
Amphipirion      GAGGAAGGAGACGACCAAAGCAGCCAATTCGAAGGCAGTGACCCCGGTGACATCATCCTC
Platichthys      GAGGACGGAGAGGACCAAACCGGCCAATTGGAAGGCGGCGACCCCAGTGACATCATCTTC
                 ***** **.** **.*.** *.****.*  **:** .* ***.* .*********** **

Black porgy      AGGCTCCTGCATCTGGCCGGCCACACTTCTCCTCATCGAATTCACCAGTGA
Haplochromis     AGGCTCCTGCATTTGGCTGGCCACAGTTCTCCTCATCGAGTCCACCAATGA
Sparus           AGGCTCCTGCATCTGGCCGGCCACACCTCTCCTCATCGAATTCACCAGTGA
Parajulis        AGGCTGCTGCATCTGGCTGGTCGCACTTCTCCTCATCGAGTCCACCAATGA
```

```
Notolabrus         AGGCTGCTGCATCTGGCTGGTCACACTTCTTCTCATCGAGTCCACCAATGA
Amphipirion        AGGTTCCTGCATCTGGCTGGCCACACTTCTCCTCACCGAATCCACCAATGA
Platichthys        AGGCTCCTGCATCTGGTCGGTCACGCTTCTCCTCATCAAAGTCACCAGTGA
                   *** * ****** ***  ** *.*.  *** **** *.*.  *****.***
```

Figure S2. Multiple alignments of the nucleotide sequences of *it* (black porgy, *Acanthopagrus schlegelii*) with other teleosts. GenBank accession numbers for the nucleotide sequences are follows: gilthead sea bream (*Sparus aurata,* FR851925 *it*), European flounder (*Platichthys flesus*, AB036518 *it*), sebase clownfish (*Amphipirion sebar*, KP260906 *it*), cichlid fish (*Haplochromis burtoni*, GQ288466 *it*), multicolour rainbowfish (*Parajulis poecilepterus*, DQ073095 *it*), spotty warsse (*Notolabrus celidotus*, MK252281 *it*). The boxes represent the ATG start codon and underlines represent the stop codon. The symbols "*", ".", and ":" denote the identity, single nucleotide variation, and more than one nucleotide variation among fish species, respectively.
