# Supplementary material for: Neurohypophysial Hormones Associated with Osmotic Challenges in the Brain and Pituitary of the Euryhaline Black Porgy, Acanthopagrus schlegelii
Source: Cells. 2021 Nov 9;10(11):3086. doi: 10.3390/cells10113086 (PMC8624723; doi:10.3390/cells10113086)
Supplement: Supplementary file 1 [file cells-10-03086-s001.zip › cells-1366984 -Fig S3-2021-10-31.pdf]

```
Black porgy    ATGGAGGACCTTTTACGCGAGCAGAATAGCTGGGCGCAGAACGTTTCATGGGGCAACTTG
Sparus         ATGGAGGACTTTTTACGCGAGCAGAATAGTTGGGCGCAGAACGTTTCATGGAGCAACTTA
Oryzias        ATGGAGGACCTTTTACTTGAGCTGGAAAGTTGGTCTCATAATGTTTCAGCCAGAAATTCC
Monopterus     ATGGAGGACATTTTACGCGAGCAGGATAACTGGGCGCAGAATATTTCATGGAACAATTCA
C.nevadensis   ATGGATGAACTTTTATCCGCACAAGATGCTTGGCTTCAGAACTTTTCCTATTGTAACTAC
Cyprinodon     ------------------------------------------------------------

Black porgy    AGCCGTGGAAATGAGAGCCATATAGGAAACACGACGGTGAATCCTCTGAAGCGAAACGAG
Sparus         AGCCGTGGAAATGAGAGTCATTTAGGAAACACGACAGTGAATCCTCTGAAGCGAAACGAA
Oryzias        AGTTGCAGGAACGAAAGTGGTGCAGAAAACAGCACAGTGAACCCTTTAAAGCGGAATGAA
Monopterus     AGTCTGGGAAATGACAGCGATTTAGGAAACACCACAGTGAATCCTTTGAAACGAAATGAA
C.nevadensis   AGTCATTTAAACAAGACTCATCCAGGGAACAACGTGGTAAACCCTTTAAAGCGAAACGAA
Cyprinodon     ---------------------------AACAACGTGGTAAACCCTTTAAAGCGAAACGAA
                                          ****  . .**.** *** *.**.**.** **.

Black porgy    GAAGTGGCCAAAGTGGAAGTTGCCGTCCTGGTGCTGGTGCTCCTGCTCGCTCTGACCGGC
Sparus         GAGGTGGCCAAAGTGGAAGTTGCCGTCCTGGTGCTGGTGCTCCTGCTCGCTCTGACCGGC
Oryzias        GAAGCAGCCAAAGTGGAGGTGACCGTCCTGGTCCTGGTGTTGTTTCTCGCTCTCATGGGC
Monopterus     GAAGTGGCCAAAGTGGAAGTTACCGTCCTGGTCCTGGTGCTGCTGCTCGCTTTGACTGGC
C.nevadensis   GAAGTGGCCAAAGTGGAAGTTACTGTCCTGGTGCTGGTGCTTTTGCTGGCTCTGACGGGT
Cyprinodon     GAAGTGGCCAAAGTGGAAGTTACTGTCCTGGTGCTGGTGCTTTTGCTGGCTCTGACGGGT
               **.* .***********.** .* ******** ****** *  * ** *** * *  **

Black porgy    AACCTGTGCGTCCTGTGGGCTATCCACTCCAGCAAGCACAGCCAGTCTCGGATGTATTTC
Sparus         AACCTGTGCGTCCTGTGGGCTATCCACGCCACCAAGCACAGCCAGTCTCGGATGTATTTC
Oryzias        AACCTGTGCGTCCTGTTGGCCATCCACACCACCAAGCACAGTCACTCCCGGATGTATTAC
Monopterus     AACCTGTGCGTCCTGTGGGCTATTAACACCACCAAGCACAGCCAGTCTCGGATGTATTAT
C.nevadensis   AACCTGTGCGTCTTGTGGGCTATCCACACCACCAAGCACAGCAAGTCTCGGATGTATTAC
Cyprinodon     AACCTGTGCGTCCTGTGGGCTATCCACACCACCAAGCACAGCAAGTCTCGGATGTATTAC
               ************ *** *** ** .** *** ********* .* ** **********:

Black porgy    TTCATGAAGCACCTGAGCATCGCGGACCTCGTCGTTGCAGTGTTTCAGGTCTTACCGCAG
Sparus         TTCATGAAGCACCTGAGCATCGCGGACCTCGTCGTTGCAGTGTTTCAGGTCTTACCGCAG
Oryzias        TTCATGAAACACCTGAGCATTGCAGACCTTGTGGTTGCAGTCTTTCAAGTTTTACCGCAG
Monopterus     TTTATGAAACACCTGAGCATCGCAGACCTTGTCGTTGCAATCTTTCAAGTTTTACCACAA
C.nevadensis   TTCATGAAGCACCTGAGCATCGCAGATCTTGTTGTGGCCGTTTTTCAGGTCCTGCCGCAA
Cyprinodon     TTCATGAAGCACCTGAGCATCGCAGATCTTGTTGTGGCCGTTTTTCAGGTCCTGCCGCAA
               ** *****.*********** **.** ** ** ** **..* *****.**  *.**.**.

Black porgy    CTCATCTGGGACATCACGTTTCGCTTCTATGGGCCGGATTTGCTGTGCAGGCTGGTGAAA
Sparus         CTCATCTGGGATATCACATTTCGCTTCTATGGACCGGATTTGCTGTGCAGGCTGGTGAAA
Oryzias        CTCATCTGGGACATCACCTTCCGCTTTTACGGACCTGACATTCTGTGCAGGCTGGTCAAG
Monopterus     CTCATTTGGGATATCACATTTCGCTTCTACGGGCCGGATTCGCTGTGCAGATTGGTCAAA
C.nevadensis   CTCATTTGGGACATAACATTTCGCTTTTACGGACCAGATATTATCTGCAGGCTCGTCAAA
Cyprinodon     CTCATTTGGGACATAACATTTCGCTTTTACGGACCAGATATTATCTGCAGGCTCGTCAAA
               ***** ***** **.** ** ***** ** **.** ** :  .* *****. * ** **.

Black porgy    TACCTCCAGGTCGTGGGCATGTTCGCGTCCACCTACATGCTCGTCCTGATGTCCATCGAC
Sparus         TACCTCCAGGTCGTGGGCATGTTCGCATCCACCTACATGCTCGTCCTGATGTCCATCGAC
Oryzias        TATCTCCAGGTCGTGGGTATGTTTGCTTCCACCTACATGCTGGTGCTGATGTCCGTCGAT
Monopterus     TACCTTCAGGTCGTGGGTATGTTTGCATCTACTTACATGCTTGTCCTGATGTCCGTCGAC
C.nevadensis   TACCTCCAAGTTGTGGGCATGTTTGCGTCCACCTACATGCTGGTCCTGATGTCCATCGAT
Cyprinodon     TATCTCCAAGTTGTGGGCATGTTTGCGTCCACCTACATGCTGGTCCTGATGTCCATCGAT
               ** ** **.** ***** ***** ** ** ** ******** ** *********.****

Black porgy    AGGTGCCTGGCGATCTGCCAGCCTCTCCGCTCCGTGCACAAGAGGAACGATCGCCTGTGC
Sparus         AGGTGCCTGGCGATCTGCCAGCCTCTCCGCTCCGTGCACAAGAGGAACGATCGCTTGTGC
Oryzias        AGGTGTTCGGCTATCTGCCTGCCATTCCGCTTCGTCAACAAGAGGAGAGACCGCATCTGC
Monopterus     AGATGCTTAGCAATTTGGCAGCCACTCCGCTCCTTGCACAAGAGAAAGGATCGCTTCTGT
C.nevadensis   AGATGTTTAGCAGTCTGCCAGCCGCTTCGCTCTGTGCACAAGGGGAAAGATCGCTTCTGT
Cyprinodon     AGATGTTTAGCAGTCTGCCAGCCGCTTCGCTCTGTGCACAAGGGGAAAGATCGCTTCTGT
               **.**   .** .* ** *:***  * ****   * .*****.*.*. ** *** * **

Black porgy    GTGATCGCCTCCTGGATGCTCAGCCTGATATTCAGCTCCCCTCAAGCCTACATATTCTCC
Sparus         GTGGTCGCCTCCTGGATGCTCAGCCTGATATTCAGCTCCCCTCAAGCCTACATATTCTCC
Oryzias        GTGATCGCCTCTTGGATGCTCAGTCTTGTTTTCAGCGCCCCGCAAGCCTACATCTTTTCT
```

```
Monopterus      GTGATCACCTCGTGGTCGCTCAGCCTGATATTTAGTACTCCTCAATTATATATATTTTCG
C.nevadensis    GTCATCGGATCCTGGATCCTCAGTCTGGTGTTCAGCACCCCACAAGCTTACATCTTTTCT
Cyprinodon      GTCATCGGATCCTGGATCCTCAGTCTGGTGTTCAGCACCCCACAAACTTACATCTTTTCT
                ** .**. .** ***:  ***** ** .* ** **  * ** ***   ** **.** **

Black porgy     TTGAAGGAGGTGGGGGACGGCGTGTATGACTGCTGGGGGGACTTTGTGCAGCCGTGGGGA
Sparus          TTGAAGGAGGTGGGGGACGGTGTGTATGACTGCTGGGGGGACTTTGTGCAGCCGTGGGGA
Oryzias         CTAAAGGAGGTCGGGAACGGGGTTTACGACTGCTGGGGGGACTTCGTGCATCCTTGGGGT
Monopterus      CTGAAGGAGGTCGGGAACGGTGTGTATGACTGCTGGGGAGACTTTGTACAGCCATGGGGT
C.nevadensis    CTCAGGGAGGTCGGGAATGGCGTCTACGACTGCTGGGGGGATTTCGTGCAACCCTGGGGT
Cyprinodon      CTCAGGGAGGTCGGGAATGGCGTCTACGACTGCTGGGGGGATTTCGTGCAACCCTGGGGT
                 * *.****** ***.* ** ** ** ***********.** ** **.** ** *****:

Black porgy     GCCAAAGCTTACATCACATGGATGAGCCTCAGCATTTACATCTTCCCAGTGGCGATTTTA
Sparus          GCCAAAGCTTACATTACATGGATGAGCCTCAGCATTTACATCTTCCCAGTGGCGATTTTA
Oryzias         GCCAAAGCGTACATCACGTGGATGAGTCTGAGCATCTACATTCTACCAGTGGCAATTCTA
Monopterus      GCAAAAGCATACGTCACATGGATGACTCTCACCATTTACTTGCTCCCAGTGGCGATTTTA
C.nevadensis    GCAAAAGCCTACATCACATGGATGAGTCTGAGCATCTACATAATTCCAGTAGCGATATTA
Cyprinodon      GCAAAAGCCTACATCACATGGATGAGTCTCAGCATCTACATAATTCCAGTAGCGATATTA
                **.***** ***.* **.*******  ** * *** ***:*  * *****.**.**: **

Black porgy     AGCGTCTGCTATGGCTTGATATGTTTCAAAATATGGCAGAATTTCAATTTGAAGACCAGA
Sparus          AGCGTCTGCTATGGCTTGATTTGGTTCAAAAAATGGCAGAATTTCAATTTGAAAACAAGA
Oryzias         GGGATCTGTTATGGCCTTATATGCTTTAAAATCTGGGAGAACATCAACATGAAAACAAGG
Monopterus      AGCACCTGCTATGGTTTAATATGTTTTAAAATATGGCAGAATTTTGATTTAAAAACCAGA
C.nevadensis    AGCATCTGCTACGGTCTGATATGCTTTAAAATATGGCAGAATATTAATATGAAAACCAAG
Cyprinodon      GGCATCTGCTACGGTCTGATATGCTTTAAAATATGGCAGAATATTAATATGAAAACCAAG
                .* . *** ** **  * **:** ** ****:.*** **** :* .* :*.**.**.*..

Black porgy     AGGGAGCACATCCTGGCCCTGACAGCGAGGCCCTCCAAGGGCGCTCTTCCCCTCACCCGT
Sparus          AGGGAGCACATCCTGGCTCTGACTGCAAGGCCCTCCAAAGGCGCTCTCCCCCTCACCCGT
Oryzias         AGAGATCGCTTTGTAGCTGTCACATCCAAAG---------GAACTCATCCACTGTCGCGT
Monopterus      AGGGAGCCATTCCTGGCTTTCACTCCCAGAGTCGCCAAAGGTGCTCATCCCCTTTCTCGC
C.nevadensis    AGGGAGCACTTTTTGGCTCTGACGCCGAGGCCGTCCAAGAGCGCACATCCACTGTCACGC
Cyprinodon      AGGGAGCACTTTTTGGCTCTGACGCCGAGGCCGTCCAAGAGCGCACATCCACTCTCACGC
                **.** * .:*  *.**  * **  * *..         * .*:*: **.** :* **

Black porgy     GTGAGCAGCGTGGGGCTCATTTCAAAAGCGAAGATCCGCACAGTGAAAATGACATTTGTG
Sparus          GTGAGCAGCGTGAGGCTCATTTCAAAAGCGAAGATCCGCACAGTGAAAATGACATTTGTG
Oryzias         GTGAGCAGCGTGAGGCTTATCTCCAAGGCCAAAATCCGCACAGTGAAGATGACATTTGTG
Monopterus      GTGAGCAGCGTGGGGCTCATTTCAAAAGCAAAGATGCGCACAGTGAAAATGACATTTGTA
C.nevadensis    GTCAGCAGCGTCAGGCTCATTTCCAAAGCCAAGATCCGCACGGTGAAAATGACATTTGTG
Cyprinodon      GTCAGCAGCGTCAGRCTCATTTCCAAAGCCAAGATCCGCACTGTGAAAATGACATTTGTG
                ** ******** .* ** ** **.**.** **.** ***** *****.***********.

Black porgy     GTGGTGGTCGCCTACATAATGTGCTGGACTCCCTTCTTCTTTGTCCAGATGTGGTCTGCA
Sparus          GTGGTGGTCGCCTACATAATGTGCTGGACTCCCTTCTTCTTTGTCCAGATGTGGTCTGCA
Oryzias         GTGGTTATTGCTTACATTGTGTGCTGGACTCCCTTTTTCTTCGTCCAGATGTGGTCTGCG
Monopterus      GTGGTCCTTGCCTATACTGTATGCTGGACTCCCTTTTTCTTTGTCCAAATGTGGTCCGCA
C.nevadensis    GTGGTTATTGCTTACATCGTATGCTGGACTCCTTTTTTCTTTGTTCAGATGTGGTCTGCA
Cyprinodon      GTGGTTATTGCTTACATCGTATGCTGGACTCCTTTTTTCTTTGTTCAGATGTGGTCTGCA
                *****  * ** ** *  .*.*********** ** ***** ** **.******** **.

Black_porgy     TGGGACCCTGCAGCACCAAGAGAAGACATGGCCTTCATCATCGCCATGTTGCTGGCCAGC
Sparus          TGGGACCCTGCAGCACCAAGAGAAGACATGGCCTTCATCATCGCCATGTTGCTGGCCAGC
Oryzias         TGGGATCCTGCTGCACCAAGAGAAGACATGGCCTTCATCATCGCGATGCTGCTGGCCAGT
Monopterus      TGGGATCCTGCTGCACCACGAGAAGACACGGCCTTCATCATCGCCATGTTGCTGGCCAGT
C.nevadensis    TGGGATCCAGCTGCACCAAGAGAAGACATGGCCTTCATCATCTCCATGTTGCTTGCTAGC
Cyprinodon      TGGGATCCAGCTGCACCAAGAGAAGACATGGCCTTCATCATCTCCATGTTGCTTGCTAGC
                ***** **:**:******.********* ************ * *** **** ** **

Black porgy     CTCAACAGCTGCTGTAACCCCTGGATCTACATGTTCTTTGCCGGTCACCTATTCCAGGAC
Sparus          CTCAACAGCTGCTGTAACCCCTGGATCTACATGTTCTTTGCCGGTCACCTGTTCCAGGAC
Oryzias         CTCAACAGCTGCTGTAACCCATGGATCTACATGTTTTTTGCTGGCCACCTCTTCCATGAC
Monopterus      CTCAACAGCTGCTGTAACCCTTGGATCTACCTGTCTTTCGCTGGTCACATGTTCCATGAC
C.nevadensis    CTTAACAGCTGCTGTAATCCCTGGATCTATATGCTTTTTGCCGGTCACCTGTTCCATGAC
Cyprinodon      CTTAACAGCTGCTGTAATCCCTGGATCTATATGCTCTTTGCCGGTCACCTGTTCCATGAC
                ** ************** ** ******** .**   ** ** ** ***.* ***** ***
```

```
Black porgy     CTGACGCGTTGCTTCTTTTGCTGCTGCAGACAGTACCTGACAGCCTCCACCTGCAGCTGC
Sparus          CTGACGCGTTGCTTCTTCTGCTGCTGCAGACAGTACCTGACAGCCTCCACCTGCAGCTGC
Oryzias         CTGATTCAGTGCTTCTTCAGCTGCTGTAGACAGTACCTGACAGACTCTTCCTGTGGCTGT
Monopterus      CTGATGCAGTGCTTCT---GCTGCTGTAGACGGTACCTGACAGACTCTTCCTGTAGCTGT
C.nevadensis    CTGATAAAGAGCTTCTGCTGCTGCTGCAGAAACTATGTTGCAGACTCATCCTGCCATGTC
Cyprinodon      CTGATAAAGAGCTTCTGCTGCTGCTGCAGAAACTATGTTGCAGACTCATCCTGCCAATGC
                ****  .. :******   ******* ***.. **  * .***.*** :****  .

Black porgy     GATCGACCGTGCCGGCACAAGAGCAGCTCCGCCACTTACGTCATCAAGAACGAGAGCAGC
Sparus          GATCGACCATGCCGGCACAAGAGCAGCTCAGCCACTTACGTCATCAAGAACGAGAGCAGC
Oryzias         GATGAACAGCGCAGGGACAAGAGCAGCTCCTCCAGCGACGTCATCAAGAATGCCAGAG--
Monopterus      GATCAACGGTGCAAGCAGAAAAGCAACCCGTCCACTTGTGTCATCAAGAACACCAGCAGT
C.nevadensis    AATCAGGAGTGCAGGCACAAGAGAGGTTCCTCCACGTTCGACATCAAAAACGGCAGCAGT
Cyprinodon      AATCAGGAGTGCAGGCACAAGAGAGGCTCCTCCACGTTCGACATCAAAAACGGCAGCAGT
                .** ..  . **..* * **.**...  *  ***     *:******.** .  **..

Black porgy     CAGAGGGGCCTCACACACACATCCAGCACGGGGGGACTTGGACACTGA
Sparus          CAGAGGAGCCTCACACACACATCCAGCACGGGGGGACTTGGACACTGA
Oryzias         -----AAGCCTCACACACACATCCAGCTTAG-----------------
Monopterus      CATCGTAGCCTCACATACATATGCAGCGCAGGGTGCCCAGGACACTAA
C.nevadensis    ATGAGGAGCTTGACTCACACATCCAGCCTGGGGGGACCCACACACTGA
Cyprinodon      ATGAGGAGCTTGACTCACACATCCAGCCTGGGGGGACCCACACACTGA
                      .** * **: *** ** ****  .*
```

Figure S3. Multiple alignments of the nucleotide sequences of *itr* (black porgy, *Acanthopagrus schlegelii*) with other teleosts. GenBank accession numbers for the nucleotide sequences are follows: gilthead sea bream (*Sparus aurata*, KC195973 *itr*), Asian swamp eel (*Monoppterus albus*, MF996357 *itr*), sheepshead minnow (*Cyprinodon variegatus*, KX061748 *itr*), amargosa pupfish (*Cyprinodon nevadensis*, GQ981415 *itr*), medaka (*Oryzias latipes*, AB646240 *itr*). The boxes represent the ATG start codon and underlines represent the stop codon. The symbols "*", ":", and ":" denote the identity, single nucleotide variation, and more than one nucleotide variation among fish species, respectively.
