# Supplementary material for: Neurohypophysial Hormones Associated with Osmotic Challenges in the Brain and Pituitary of the Euryhaline Black Porgy, Acanthopagrus schlegelii
Source: Cells. 2021 Nov 9;10(11):3086. doi: 10.3390/cells10113086 (PMC8624723; doi:10.3390/cells10113086)
Supplement: Supplementary file 1 [file cells-10-03086-s001.zip › cells-1366984 -Fig S4-2021-10-31.pdf]

```
Black porgy    ATGCCTCACTCCTTGTCCCCCCTGTGCGTCCTGGGACTCAT---CGCGCTCTCCTCCGCC
Sparus         ATGCCTCACTCCTTGTTCCCCCTGTGCGTCCTGGGACTCCT---TGCGTTCTCCTCTGCC
Paralicthys    ATGCCTCACTCCATGTTCCCGCTGTGCGTCCTGGGATTCCT---CGCCCTCTCCTCCGCC
Platichthys    ATGCCTCACTCCATGTTCCCGCTGTGCGTCCTGGGATTCCT---CGCCCTCTCCTCCGCC
Halichoeres    ATGCCACACTCCGTGATCCCTCTGTGTGTCCTGGGAATCCTTCTCGCGCTCTCCTCGGCT
Epinephelus    ATGCCTCACTCCATGTTCCCCCTGTGCGTCCTGGGACTCCT---CACGCTCTCATCCGCC
               *****:****** **: *** ***** ********* **.*    .*  ****.** **

Black porgy    TGCTACATCCAGAACTGCCCCCGAGGAGGGAAGCGGGCGCTGCCGGAGGCTGGGACCAGA
Sparus         TGCTACATCCAGAACTGCCCCCGAGGAGGGAAGCGGGCGCTGCCAGAGGCTGGGATCAGA
Paralicthys    TGCTACATCCAGAACTGTCCCCGGGGGGGGAAGCGAGCGCTGCCGGACACCGGCATCAGA
Platichthys    TGCTACATCCAGAACTGCCCCCGGGGAGGGAAGCGAGCGCTGCCGGACACTGGCATCAGA
Halichoeres    TGTTACATCCAGAACTGCCCCCGAGGAGGGAAACGCGCACTGCCGGAGACTGGGCTCAGA
Epinephelus    TGTTACATCCAGAACTGCCCGAGAGGAGGGAAGCGAGCGCTGCCGGAGACTGGGATCAGA
               ** ************** ** .*.**.*****.** **.*****.** .* ** . ****

Black porgy    CAGTGCATGTCGTGTGGCCCCAGAGACAGGGGCCGCTGCTTCGGCCCCAACATCTGCTGC
Sparus         CAGTGCATGTCGTGTGGCCCCAGAGACAGGGGCCACTGTTTCGGCCCCAACATCTGCTGC
Paralicthys    CAGTGCATGTCCTGTGGCCCCGGAGACAGGGGCCGCTGCTTCGGCCCCAGTATCTGCTGC
Platichthys    CAGTGCATGCCCTGTGGCCCCGGGGACAGGGGCCGCTGCTTCGGCCCCGGTATCTGCTGC
Halichoeres    CAGTGCATGCCGTGTGGCCCCAGAGACAAGGGCCGCTGCTTTGGCCCAAATATCTGCTGT
Epinephelus    CAGTGCATGTCTTGTGGCCCCGGAGACAGGGGCCGCTGCTTCGGCCCCAGCATCTGCTGC
               ********* * *********.*.****.*****.*** ** *****... ********

Black porgy    GGGGAGGGCCTCGGCTGTCTGCTGGGCTCCCCGGAAACAGCTCACTGCGTGGAGGAGAAC
Sparus         GGGGAGGGCCTCGGCTGTCTGCTGGGCTCCCCGGAAACAGCTCACTGTGTGGAGGAGAAC
Paralicthys    GGGGAGGGTCTGGGCTGCCTGCTGGGCTCCCCAGAGACAGCTCACTGTGTGGAGGAGAAC
Platichthys    GGGGAGGGTCTTGGCTGCCTGCTGGGCTCCCCAGAGACAGCTCACTGTGTGGAGGAGAAC
Halichoeres    GGGGAGGGCCTTGGCTGTTTGCTGGGCTCCCCAGAAACAGCTCACTGTGTGGAAGAGAAC
Epinephelus    GGGGAGGGCCTCGGCTGCCTGCTGGGCTCCCCTGAAACAGCTCACTGTGTGGAGGAGAAC
               ******** ** *****  ************* **.*********** *****.******

Black porgy    TACCTGCTCACCCCCTGCCAGGCGGGAGGGAGACCCTGTGGCTCTGAAGGAGGACGCTGC
Sparus         TACCTGCTCACCCCCTGCCAGGCGGGAGGGAGACCCTGTGGCTCTGAAGGAGGACGCTGC
Paralicthys    TACCTGCTCACCCCCTGCCAGGCAGGAGGGAGACCCTGTGGATCTGAGGGAGGACACTGT
Platichthys    TACCTGCTCACCCCCTGCATGGCAGGAGGGAGACCCTGTGGATCTGAGGGAGGACGCTGT
Halichoeres    TACCTGCTCACCCCCTGCCAGGCAGGAGGGAGACCCTGTGGATCTGAGGGAGGGCGCTGT
Epinephelus    TACCTGCTCACGCCCTGCCAGGCAGGAGGGAGACCTTGTGGCTCTGAGGGAGGACGCTGC
               *********** ******.:***.*********** *****.*****.*****.*.***

Black porgy    GCTGCTTCAGGACTCTGCTGTAACTCAGAGAGCTGCACGGTGGACTCTGACTGTCTCGGG
Sparus         GCTGCTTCAGGACTCTGCTGTAACTCAGAGAGCTGTACGGTGGACTCTGACTGCCTTGGG
Paralicthys    GCAGCTTCAGGACTCTGCTGTAACTCAGAGAGCTGTGCAGTTGACTCTGACTGCCTGGCA
Platichthys    GCGGCATCAGGACTCTGCTGTAACCCAGAGAGCTGTGCGGTAGACTCTGACTGCCTGGCG
Halichoeres    GCTGCCTCAGGAGTCTGCTGTAACTCAGAGAGCTGTGCGGTGGACTCTGACTGCCTGGGG
Epinephelus    GCTGCTTCAGGACTCTGCTGTAACTCAGAGAGCTGCATGGTGGACCCTGACTGCCTGGGG
               ** ** ****** *********** ********** . .** *** ******* ** * .

Black porgy    GAGGTTGAGGCCTCAGACCCGTCTGACAGCTCTGTGGGGAGTTCGCCTGCAGAGCTGCTG
Sparus         GAGGTTGAGGCCTCAGACCCGTCCGACAGCTCTGCGGGGAGCTCGCCTGCAGAGCTGCTG
Paralicthys    GAGACCGAGGCCTCAGATCCGGGCCACGGCGCCGCAGGGAGCTTCCCGGCAGAGCTGCTG
Platichthys    GAGATCGAGGCCTCAGATCCGGGCCACGGCGCCGGAGGGAGCTCACCTGCAGCGCTGCTG
Halichoeres    GAGATGGAAGCGTCAGATCAGGCCGACAGCTCTGCAGGGAGCTCGCCTGCGGAGCTGCTG
Epinephelus    GACACCGAGGCCTCAGATCCGGCTCACGGCTCTGCAGGGAGCTCACCTGCAGAACTGCTG
               **  . **.** ***** *.*    **.** * * .***** *  ** **.*..******

Black porgy    CTGCGCCTGCTACATGTGGCCACCAGAGGACAGACCGAGTACTGA
Sparus         CTGCGCCTGCTACATGTGGCCACCAGAGGACAGACCGAGTACTGA
Paralicthys    CTCCGCCTGCTGCATGTGACGGCCAGAGGACAGACCGAGTACTGA
Platichthys    CTCCGCCTGCTGCATGTGACGGCCAGAGGACAGACCGAGTACTGA
Halichoeres    CTGCGGCTGCTACATGTGGCCACAAGAGGACAGACCGAGTACTGA
Epinephelus    ATGCGCCTGCTGCACGTAGCCACCAGAGGACAAACCGAGTACTGA
               .* ** *****.** **..* .*.********.************
```

Figure S4 Multiple alignments of the nucleotide sequences of *avt* (black porgy, *Acanthopagrus schlegelii*) with other teleosts. GenBank accession numbers for the nucleotide sequences are

follows: gilthead sea bream (*Sparus aurata,* FR851924), orange spotted grouper (*Epinephelus coioides*, GU831571), European flounder (*Platichthys fleus*, AB036517), Chinese warasse (*Halichoeres tenuispinis*, GU212654), olive flunder (*Paralicthys olivaceus*, AB856411). The boxes represent the ATG start codon and underlines represent the stop codon. The symbols "*", ".", and ":" denote the identity, single nucleotide variation, and more than one nucleotide variation among fish species, respectively.
