# Supplementary material for: Neurohypophysial Hormones Associated with Osmotic Challenges in the Brain and Pituitary of the Euryhaline Black Porgy, Acanthopagrus schlegelii
Source: Cells. 2021 Nov 9;10(11):3086. doi: 10.3390/cells10113086 (PMC8624723; doi:10.3390/cells10113086)
Supplement: Supplementary file 1 [file cells-10-03086-s001.zip › cells-1366984 -Fig S5-2021-10-31.pdf]

```
Black porgy    ATGCACACTCCCGACTATGCGCTTGTCCTGAGCGGAGGGAACAGGTCTCTGGTCTTCAGT
Oryzias        ATGTACACTCTCTCCAGCGTGCTGCTCTTGAGCGGAGAGAACTGTTCTCTGGATTCCAGT
Epinephelus    ------------------------------------------------------------
Amphiprion     ------------------------------------------------------------
Halichores     ------------------------------------------------------------
Paralicthys    ------------------------------------------------------------

Black porgy    CCCACTGATGACTTCACGATGGGAACACCTGGGAACGACAGCGTCCACCACAACGGATCC
Oryzias        TTGTCCAGTGACCTCATGATGGGAACGTCTGGAAACGGCACCGCGAACTCGAACGGATCC
Epinephelus    ------------------------------------------------------------
Amphiprion     ------------------------------------------------------------
Halichores     ------------------ATGGGGACTCCTGGGAACGACACGGTCTACCCCAACGGGTCT
Paralicthys    ------------------ATGGAGAAGCCTGGAAACATCACCCTCCACCCGAACGGCTCC

Black porgy    GATCCTTTTGCGCGGAACGAAGAGGTGGCCCAAATCGAGATCATGGTGCTGAGCATCACT
Oryzias        GACCCGTTTGGCAGGAATGAGGAGGTGGCCCAAATCGAGATCATGGTCCTGAGCATCACC
Epinephelus    GACCCGTTTGGGCGGAACGAGGAGGTGGCCCAGATCGAGATAATGGTCGTGAGCATCACC
Amphiprion     ------------------------------------------------------------
Halichores     GATCCGTACGCGCGGAACGAGGAGGTGGCGCAGATGGAGATCCTGGTCCTGAGCATCACC
Paralicthys    GATCCGTTCGCGAGGAACGAGGAGGTGGCCCAGATCGAGATCATGGTGCTGTGCATCACT

Black porgy    TTCGTGGTCGCTGTGGTCGGCAACGTGAGCGTCCTGCTGGCAATGTACAACACGAAGAAG
Oryzias        TTTGTGGTTGCAGTGATTGGAAACGTGAGCGTCCTTTTGGCGATGTACAACACCAAGAAG
Epinephelus    TTCGTGGTTGCTGTGATTGGGAATGTGAGCGTCCTGCTGGCAATGTACAACACAAAGAAG
Amphiprion     ------------------------------------------------------------
Halichores     TTCGTGGTGGCTGTGATCGGGAACGTGAGCGTCCTGCTGGCGATGTACAACACCAAGAAG
Paralicthys    TTGGTGGTCGCCGTGATCGGAAACGTGAGCGTCCTTCTGGCGATGTACAACACCAAGAAG

Black porgy    AAGATGTCGCGGATGCACCTCTTCATCAAACACCTCAGCCTGGCTGACCTGGTGGTCGCC
Oryzias        AAGATGTCGCGGATGCACCTCTTCATCAAACACCTCAGCCTGGCTGATCTGGTGGTCGCC
Epinephelus    AAGATGTCGCGGATGCACCTTTTCATCAAGCACCTCAGCTTGGCTGACCTGGTGGTCGCC
Amphiprion     ------------------------------------------------------------
Halichores     AAGATGTCCCGGATGCACCTCTTCATCAAACACCTGAGCCTGGCCGACCTGGTGGTCGCC
Paralicthys    AAGATGTCGCGCATGCACCTCTTCATCAAACACCTGAGCCTGGCTGACCTGGTGGTCGCC

Black porgy    TTCTTCCAGGTGCTGCCGCAGCTCTGCTGGGAGATCACCTTCCGCTTCTACGGTCCGGAC
Oryzias        TTCTTCCAGGTGCTACCGCAGCTCTGCTGGGAGATCACCTTCCGCTTCTACGGCTCAGAC
Epinephelus    TTCTTCCAGGTGCTGCCGCAGCTCTGCTGGAAGATCACCTACCGCTTCTACGGTTCAGAC
Amphiprion     ------------------------------------------------------------
Halichores     TTCTTCCAGGTGCTGCCGCAGCTCTGCTGGGAGGTCACCTTCCGCTTCTACGGCCCGGAC
Paralicthys    TTCTTCCAGGTGCTGCCGCAGCTCTGCTGGAAGGTTACCTTCCGCTTCTATGGTCCGGAC

Black porgy    TTTCTCTGCAGGATAGTGAAGCACCTGCAGGTGATGGGGATGTTCGCGTCCACCTACATG
Oryzias        TTTCTGTGCAGGATTGTAAAGCACCTCCAGGTGATGGGAATGTTTGCGTCCACCTATATG
Epinephelus    TTTCTCTGCAGGGTCGTCAAGCACCTCCAGGTGATGGGGATGTTCGCGTCCACTTACATG
Amphiprion     ------------------------------------------------------------
Halichores     TTTCTGTGCCGGATCGTCAAACACCTCCAGGTGATGGGGATGTTCGCGTCCACCTACATG
Paralicthys    TTTCTATGCAGGATCGTGAAGCACCTCCAGGTGACGGGGATGTTTGCGTCCACCTACGTG

Black porgy    ATGGTGATGATGACCCTGGACCGTTACATCGCCATCTGCCACCCCCTGAAAACCCTCCAG
Oryzias        ATGGTGATGATGACTTTGGACCGTTACATCGCCATCTGCCACCCTCTGAAAACCCTCCAG
Epinephelus    ATGGTGATGATGACCGTGGACCGTTACATTGCCATCTGCCACCCTCTGAAAACCCTGCAG
Amphiprion     ------------------------------------------------------------
Halichores     ATGGTGATGATGACCCTGGACCGGTACATCGCCATCTGCCACCCTCTGAAGACCCTGCAG
Paralicthys    ATGGTGATGATGACCCTTGACCGCTACATCGCCATCTGCCACCCGCTGAAAACCCTCCAT
```

```
Black porgy     CAGCCCACCCAGCGCTCCTACATCATGATAATCTCCACGTGGATGTGCAGCCTGGTGTTA
Oryzias         CAGCCCACCAAGCGCTCCTACATCATGATTACCTCCACGTGGATGTGCAGCCTGGTGCTC
Epinephelus     CAGCCCACCCAGCGCTCCTACATCATGATCATCTCCACGTGGATGTGCAGCCTGGTGTTC
Amphiprion      ------------------------------------------------------------
Halichores      CAGCCCACGCAGCGCGCCTACATCATGATCGTGTCCACGTGGATGTGCAGCCTGGTGCTC
Paralicthys     CAGCCCACTCAGCGCTCCTACATCATGATTGTCTCCACGTGGATGTGCAGCCTGGTGTTC

Black porgy     AGCACCCCGCAGTACTTCATCTTCTCCCTGAGCGAGATCAAGAACGGCTCGGATGTTTAC
Oryzias         AGCACCCCGCAGTACTTCATCTTCTCCCTGAGTGAGATCAAAAACGGCTCGGACGTGTAC
Epinephelus     AGCACCCCGCAGTACTTCATCTTCTCCCTGAGCGAGATCAAGAACGGCTCGAAAGTAAAC
Amphiprion      --------------------------------CGAGATCGAGAACGGCTCGGACGTCTAC
Halichores      AGCACCCCGCAGTACTTCATCTTCTCCCTGAGCGAGATCAAGAACGGGTCCGAGGTCTAC
Paralicthys     AGCACGCCGCAGTACTTCATCTTCTCCCTGAGCGAGATCAAGAACGGCTCGAAAGTCTAC
                                                 ******.*.***** ** .* ** :**

Black porgy     GACTGCTGGGCGTACTTCATCGAGCCATGGGGCGCCAAGGCGTATATCACCTGGATAACC
Oryzias         GACTGCTGGGCGCACTTCATCCAGCCCTGGGGCGCAAAGGCGTATATCACCTGGATAACA
Epinephelus     GACTGCTGGGCGCACTTTATCGAGCCGTGGGGCGCCAAGGCGTACATCACCTGGATAACT
Amphiprion      GATTGCTGGGCGCACTTCATCGAGCCCTGGGGCGCCAAGGCGTACATCACCTGGATAACC
Halichores      GACTGCTGGGCGCACTTCATCGAGCCGTGGGGCGCCAAGGCGTACATCACCTGGATCACC
Paralicthys     GACTGCTGGGCGCACTTCATCGAGCCCTGGGGCGCCAGGGCGTACATCACCTGGATCACC
                ** ********* **** *** **** ********.*.****** ***********.**

Black porgy     GTGGGCATCTTCCTCGTGCCCGTGGTCATTCTCATGATGTGCTACGGGTTCATCTGCCAC
Oryzias         GTGGGCATCTTCCTCGTCCCTGTAGTCATCCTCATGTTGTGTTACGGGTTCATCTGCCAC
Epinephelus     GTGGGCATCTTCCTCGTGCCCGTGGTCATTCTCGTGATGTGCTACGGGTTCATCTGCCAC
Amphiprion      GTGGGCATCTTCCTCGTGCCCGTTGTCATTCTCATGATGTGCTACGGGTTCATCTGCCAC
Halichores      GTGGGCATCTTCCTCGTGCCTGTGGTCATCCTCGTGCTGAGCTACGGCTTCATCTGCCAC
Paralicthys     GGCGGGATCTTCCTGGTGCCCGTGGTCATCCTTGTGATGTGCTACGGCTTCATCTGCCAC
                *  ** ******** ** ** ** ***** ** .** **:* ***** ************

Black porgy     AGCATATGGAAAAATATCAAATACAAGAAAAGGAAAACGATGGCCGGCGCTGCCAGCAAG
Oryzias         AGTATATGGAAAAACATAAAGTACAAGAAAAGAAAAACGACGGCTGGAGCATCAAATAAG
Epinephelus     AGCATATGGAATAATATCAAATACAAGAAAAGGAAATCGACGGCTGGTGCTGCGAACAAG
Amphiprion      ACTATATGGAAAAATATCAAATTCAAGAAAAGGAAAACGATGTCTGGGGCTGCGAGCAAG
Halichores      AGCATTTGGAAAAACATCAAATACAAGAAGCGGAAAACGACCGGAGGAGCCGCTGCGAAG
Paralicthys     AGCATATGGAGAAATATCAAATACAAGAAAAGGATAACGATGGCTGGCGCTGTGAGCAAG
                *  **:****.:** **.**.*:******..*.*:*:***     ** **    .  ***

Black porgy     AACGCGATAATCGGGAAGAATTCAGTCAGCAGTGTTACAACGATATCCAGAGCCAAGCTG
Oryzias         AACGGGCTCATCGGGAAGAACTCCGTCAGTAGCGTCACCACCATCTCCAGAGCCAAACTC
Epinephelus     AACGGGCTGATTGGGAAGCATTCAGTCAGCAGCATTACAACGATATCCAGAGCCAAACTG
Amphiprion      AATGGATTGATTGGAAAGAATTCAGTCAGCAGCGTCACAACTATATCAAGAGCCAAACTG
Halichores      AGCGGGCTGATCGGGAAGAACTCAGTCAGCAGCGTCACCACCATCTCCAGAGCCAAGCTG
Paralicthys     AACGGGTTGATTGGAAAGAATTCAGTCAGCAGCGTCACGACCATATCGAGAGCCAAGCTG
                *. * . * ** **.***.* **.***** ** .* ** ** **.** ********.**

Black porgy     AGGACTGTCAAAATGACTTTTGTGATAGTTTTGGCGTACATTATCTGCTGGGCGCCGTTT
Oryzias         CGCACGGTTAAAATGACTTTTGTGATCGTTTTGGCCTACATTGTTTGCTGGTCGCCGTTT
Epinephelus     AGGACTGTAAAAATGACTTTTGTGATAGTTTTGGCGTACATCGTGTGCTGGGCGCCGTTT
Amphiprion      AGGACCGTTAAAATGACTTTCGTGATAGTTCTGGCGTACATTATCTGCTGGGCCCCGTTT
Halichores      AGGACTGTCAAAATGACTTTCGTGATCGTCCTGGCCTACATCGTGTGCTGGGCGCCGTTC
Paralicthys     CGGACTGTTAAGATGACTTTTGTGATAGTTTTGGCGTACATCATTTGTTGGGCGCCGTTT
                .* ** ** **.******** *****.**  **** ***** .* ** *** * *****

Black porgy     TTCATCGTGCAGATGTGGTCGGTGTGGGATGAGAACTTCCAGTGGGCTGATTCTGAGAAC
Oryzias         TTCATAGTGCAAATGTGGTCTGTGTGGGATGAAAACTTCTTGTGGGACGATTCTGACAAC
Epinephelus     TTCACAGTGCAGATGTGGTCTGTGTGGGATAAAAACTTCCAGTGGGTTGATTCTGAGAAC
Amphiprion      TTCATCGTGCAGATGCGGTCTGTGTGGGATGAAAACTTCCAGTGGGCTGATTCTGTGGAC
Halichores      TTCATCGTTCAGATGTGGTCCGTGTGGGACGAGAACTCCCAGTGGGCCGAATCCGAGAAC
Paralicthys     TTCACCGTGCAGATGTGGTCGGTGTGGGATGAAAACTTCATGTATGCTGATTCTGAGAAC
                **** .** **.*** **** ******** .*.**** * :**. *  **:** *: .**

Black porgy     ACAGCAGTGACTCTGTCTGCGCTCCTTGCCAGTCTCAACAGCTGCTGTAACCCGTGGATA
Oryzias         ACAGCAGTGACTCTGTCGGCACTCCTCGCCAGTCTCAACAGCTGCTGCAACCCGTGGATT
Epinephelus     ACAGCAGTGACTCTGTCTGCGCTTCTTGCCAGTCTCAACAGCTGCTGTAACCCGT-----
Amphiprion      ACAGCAGTGACTCTGTCTGCGCTCCTTGCCAGT---------------------------
```

```
Halichores       ACGGCGGTGACTCTGTCTGCGCTGCTTGCCAGTCTCAACAGCTGCTGCAACCCGTGGATA
Paralicthys      ACAGCAGTGACTCTTTCTGCGCTCCTTGCCAGTCTCAACAGCTGCTGCAACCCGTGGATA
                 **.**.******** ** **.** ** ******

Black porgy      TACATGATCTTCAGCGGTCACCTCCTCCAGGATTTCGTGCACTGCTTCTCGTGCTGCCTC
Oryzias          TACATGATCTTCAGCGGCCATCTCCTGCAAGATTTTGTGCACTGCTTCTCCTGCTGCCGG
Epinephelus      ------------------------------------------------------------
Amphiprion       ------------------------------------------------------------
Halichores       TACATGATCTTCAGCGGTCACCTCCTCCAGGATTTCATGCACTGCTTCTCCTGCTGCCTC
Paralicthys      TACATGATCTTCAGCGGCCACCTCCTGCAGGATTTCGTGCACTGCTTCTCCTGTTGCCAC

Black porgy      AGGATGAACACTGACTTCAAGAAGGAGGACTCGGACAGCAGTATCCGCAGGACAACGCTG
Oryzias          AGGGTAAACGCT---GATAAGAAAGAGGACTCGGACAGCAGCATCAGACGGACCACGTTG
Epinephelus      ------------------------------------------------------------
Amphiprion       ------------------------------------------------------------
Halichores       AGGCTCAACCCCGACTACAAGAAGGAGGACTCGGACAGCAGCCTCCGCAGGACCACGCTG
Paralicthys      AGAGCAAACACGGACTTCAAGAAGGAGGACTCGGACAGCAGCATCCGCAGGACGACGCTG

Black porgy      CTGACTAAGATGACCAATCGGAGCCCAACAGGCAGCTCCGCAAACTGGAGAGAGCTGGAC
Oryzias          CTGACTAAGATGACCAACCGGAGCCCCACAGGCAGCACTGGCAACTGGAAGGAGCTGGAC
Epinephelus      ------------------------------------------------------------
Amphiprion       ------------------------------------------------------------
Halichores       CTCACCAAGATGACCAACCGCAGCCCGACGGGGAGCACGGGGAACTGGAGAGAGTTTGAC
Paralicthys      CTGACTAAGATGACCAACCGCAGCCCCACAGGCAGCACTGGCAACTGGAGAGAGTTGGAC

Black porgy      AATTCTCCCAAATCCTCCATTCAGCCAGAGGAAAATAACAGCTAG---------------
Oryzias          AATTCTCCCAAAAACTCTGCTCAGGCAGAGTAAACAGCAGAGCAGAGACATCATAAAATG
Epinephelus      ------------------------------------------------------------
Amphiprion       ------------------------------------------------------------
Halichores       AACTCACCCAAGACCTCGATTCAGGCTGATTGA---------------------------
Paralicthys      AACTCTCCCAAGACCTCCATTCAGATGGAGTAA---------------------------
```

Figure S5. Multiple alignments of the nucleotide sequences of *avtr* (black porgy, *Acanthopagrus schlegelii*) with other teleosts. GenBank accession numbers for the nucleotide sequences are follows: orange spotted grouper (*Epinephelus coioides*, GU929704), clown anemonefish (*Amphiprion ocellaris*, AB597955), olive flounder (*Paralicthys olivaeus,* EF451960), threespot wrasse (*Halichores trimaculatus*, AB642257), medaka (*Oryzias latipes*, NM001308987). The boxes represent the ATG start codon and underlines represent the stop codon. The symbols "*", ".", and ":" denote the identity, single nucleotide variation, and more than one nucleotide variation among fish species, respectively.
