# Supplementary material for: Neurohypophysial Hormones Associated with Osmotic Challenges in the Brain and Pituitary of the Euryhaline Black Porgy, Acanthopagrus schlegelii
Source: Cells. 2021 Nov 9;10(11):3086. doi: 10.3390/cells10113086 (PMC8624723; doi:10.3390/cells10113086)
Supplement: Supplementary file 1 [file cells-10-03086-s001.zip › supplementary Fig S1-2021-11-11-new.pdf]

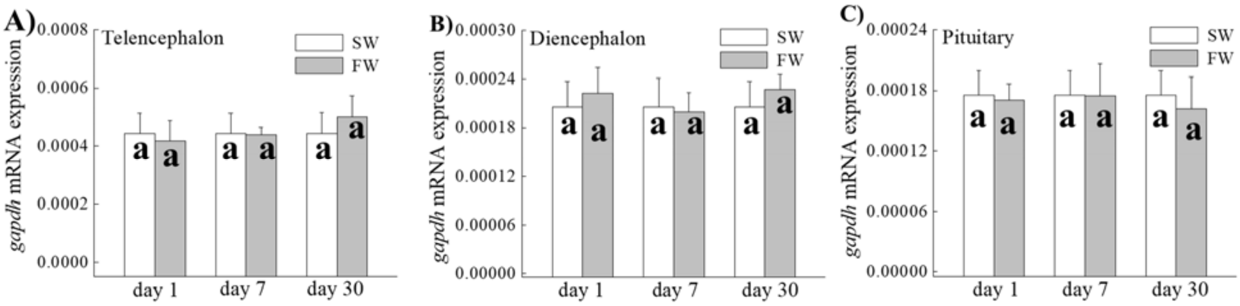

Figure S1. The expression (n = 8 in each data) of internal control gene *gapdh* (*glyceraldehyde 3-phosphate dehydrogenase,* GenBank accession no: DQ399798) in the telencephalon (A), diencephalon (B), and pituitary (C) of black porgy acclimated to seawater (SW) and freshwater (FW). No significant difference (denoted with a) of *gapdh* transcripts was found between SW and FW fish. No significant changes (denoted with a) of *gapdh* transcripts were observed on day 1, 7, and 30.
